# MicroRNA Discovery and Analysis of Pinewood Nematode *Bursaphelenchus xylophilus* by Deep Sequencing

**DOI:** 10.1371/journal.pone.0013271

**Published:** 2010-10-12

**Authors:** Qi-Xing Huang, Xin-Yue Cheng, Zhen-Chuan Mao, Yun-Sheng Wang, Li-Lin Zhao, Xia Yan, Virginia R. Ferris, Ru-Mei Xu, Bing-Yan Xie

**Affiliations:** 1 College of Life Sciences, Beijing Normal University, Beijing, China; 2 Institute of Vegetables and Flowers, Chinese Academy of Agricultural Sciences, Beijing, China; 3 Hunan Province Key Laboratory of Crop Germplasm Innovation and Utilization, Hunan Agricultural University, Changsha, China; 4 State Key Laboratory of Integrated Management of Pest Insects and Rodents, Institute of Zoology, Chinese Academy of Sciences, Beijing, China; 5 Department of Entomology, Purdue University, West Lafayette, Indiana, United States of America; Deutsches Krebsforschungszentrum, Germany

## Abstract

**Background:**

MicroRNAs (miRNAs) are considered to be very important in regulating the growth, development, behavior and stress response in animals and plants in post-transcriptional gene regulation. Pinewood nematode, *Bursaphelenchus xylophilus*, is an important invasive plant parasitic nematode in Asia. To have a comprehensive knowledge about miRNAs of the nematode is necessary for further in-depth study on roles of miRNAs in the ecological adaptation of the invasive species.

**Methods and Findings:**

Five small RNA libraries were constructed and sequenced by Illumina/Solexa deep-sequencing technology. A total of 810 miRNA candidates (49 conserved and 761 novel) were predicted by a computational pipeline, of which 57 miRNAs (20 conserved and 37 novel) encoded by 53 miRNA precursors were identified by experimental methods. Ten novel miRNAs were considered to be species-specific miRNAs of *B. xylophilus*. Comparison of expression profiles of miRNAs in the five small RNA libraries showed that many miRNAs exhibited obviously different expression levels in the third-stage dispersal juvenile and at a cold-stressed status. Most of the miRNAs exhibited obviously down-regulated expression in the dispersal stage. But differences among the three geographic libraries were not prominent. A total of 979 genes were predicted to be targets of these authentic miRNAs. Among them, seven heat shock protein genes were targeted by 14 miRNAs, and six FMRFamide-like neuropeptides genes were targeted by 17 miRNAs. A real-time quantitative polymerase chain reaction was used to quantify the mRNA expression levels of target genes.

**Conclusions:**

Basing on the fact that a negative correlation existed between the expression profiles of miRNAs and the mRNA expression profiles of their target genes (*hsp*, *flp*) by comparing those of the nematodes at a cold stressed status and a normal status, we suggested that miRNAs might participate in ecological adaptation and behavior regulation of the nematode. This is the first description of miRNAs in plant parasitic nematodes. The results provide a useful resource for further in-depth study on molecular regulation and evolution of miRNAs in plant parasitic nematodes.

## Introduction

MicroRNAs (miRNAs) are single-stranded RNAs of ∼22nt in length that are generated from endogenous hairpin-shaped transcripts (reviewed in [1]). They function as guide molecules in post-transcriptional gene regulation by base-pairing with the target mRNAs, usually in the 3′ untranslated regions (UTRs), or, in a few cases, with target sites in the coding regions [2]. Since the original description of *lin-4* in 1993 [3], [4], miRNAs have been found in a wide range of eukaryotic organisms ranging from sponges to mammals [5]. So far, more than ten thousand miRNAs of 115 species have been registered in miRBase (ftp://mirbase.org/pub/mirbase/, release 14.0, September 2009). It was reported that miRNAs regulate at least 10% of *Caenorhabditis elegans* genes through conserved interactions and a number of nematode miRNAs regulate biological processes by targeting functionally related genes [6]. Computational study has suggested that over one third of human genes are possibly targeted by miRNAs, and most of which are transcriptional and developmental factors [7]. Besides, abnormal expressions of miRNA genes may cause human disease, dramatic phenotype changes or death (reviewed in [8], [9]). Furthermore, stress-inducible miRNAs were discovered in response to specific conditions [10], [11]. MiRNAs act as rheostats to make fine-scale adjustments to protein output [12] . So, miRNAs have received much attention by biological scientists in recent years. MiRNAs and other small noncoding RNAs, e.g. small interfering RNAs (endo-siRNAs) and piwi-interacting RNAs (piRNAs) have been intensely studied in *Caenorhabditis elegans* [13]–[16] and closely related species [17]. Recently, miRNAs were also reported in the human filarial parasite *Brugia malayi* [18] and the necromenic nematode *Pristionchus pacificus* [17]. Nevertheless, there are no reports about miRNAs and other small RNAs in plant parasitic nematode.

The pinewood nematode, *Bursaphelenchus xylophilus* (Steiner et Burhrer), is a successful invasive plant parasitic nematode, which kills living pine trees and causes many thousands of pine trees to die in Asia [19]–[21]. This nematode species is believed to be native to North America, and usually only damages exotic pine trees there. Two morphological forms exist in the native region, i.e., the ‘R’ form with a round female tail and the ‘M’ form with a mucronate female tail [22]–[25]. It was reported that the pathogenicity of the two forms differs. The ‘R’ form is strongly pathogenic, and the nematodes now present in Asia are the ‘R’ form. The pathogenicity of the ‘M’ form is weaker than that of the ‘R’ form [23], [25]. This nematode has complex and intriguing life cycles, including a propagative cycle and a dispersal cycle. Under favorable conditions (suitable moisture, food and temperature), the nematode development follows a reproductive pathway in host trees. When within-wood conditions deteriorate, the nematode development switches from the second-stage propagative larvae (L2) to the third-stage dispersal juvenile (J3)[26], [27]. When the larvae of vector beetle (*Monochamus* spp.) pupate, the third-stage dispersal juveniles aggregate on the wall of the pupal chamber. Then, the juveniles molt to the fourth-stage dauer juveniles at the time of the vector beetle eclosion, and they enter into the respiratory system of the newly eclosed adult beetle for dispersal. It was suggested that chemical substances play an important role during the process [26]. Moreover, the pinewood nematode has a widespread ecological adaptation, and an extensive distribution range [20], [28]. Cold and heat tolerance tests showed that the nematode has a strong tolerance to temperature stresses [21].

Because miRNAs regulate gene expression and play important roles in the development, metabolism and behavior of animals (reviewed in [29]), identification of miRNAs and other small RNAs could be a critical step to facilitate our understanding of the molecular regulation mechanisms of the nematode. In this study, by constructing five specific small RNA libraries (the Chinese, American and Canadian nematodes, third-stage dispersal juveniles and cold stressed nematodes) and sequencing with the Illumina/Solexa deep sequencing technology, miRNA candidates were predicted by a computational pipeline and 57 miRNAs were verified successfully by experimental methods. We compared miRNA expression profiles in the five small RNA libraries and predicted their potential target genes. We especially paid more attention to two sorts of target genes: heat shock protein genes (*hsps*) and FMRFamide-like neuropeptides genes (*flps*). The former are related to environmental stress and the latter are related to neuronal sensitivity and modulate sensory and motor functions. We explored the roles of miRNAs in ecological adaptation of the pinewood nematode. Moreover, as miRNAs are evolutionarily conserved across species, our results may become a useful resource for miRNA studies in other plant parasitic nematodes.

## Results

### An overview of small RNA sequencing results

**Figure 1:**
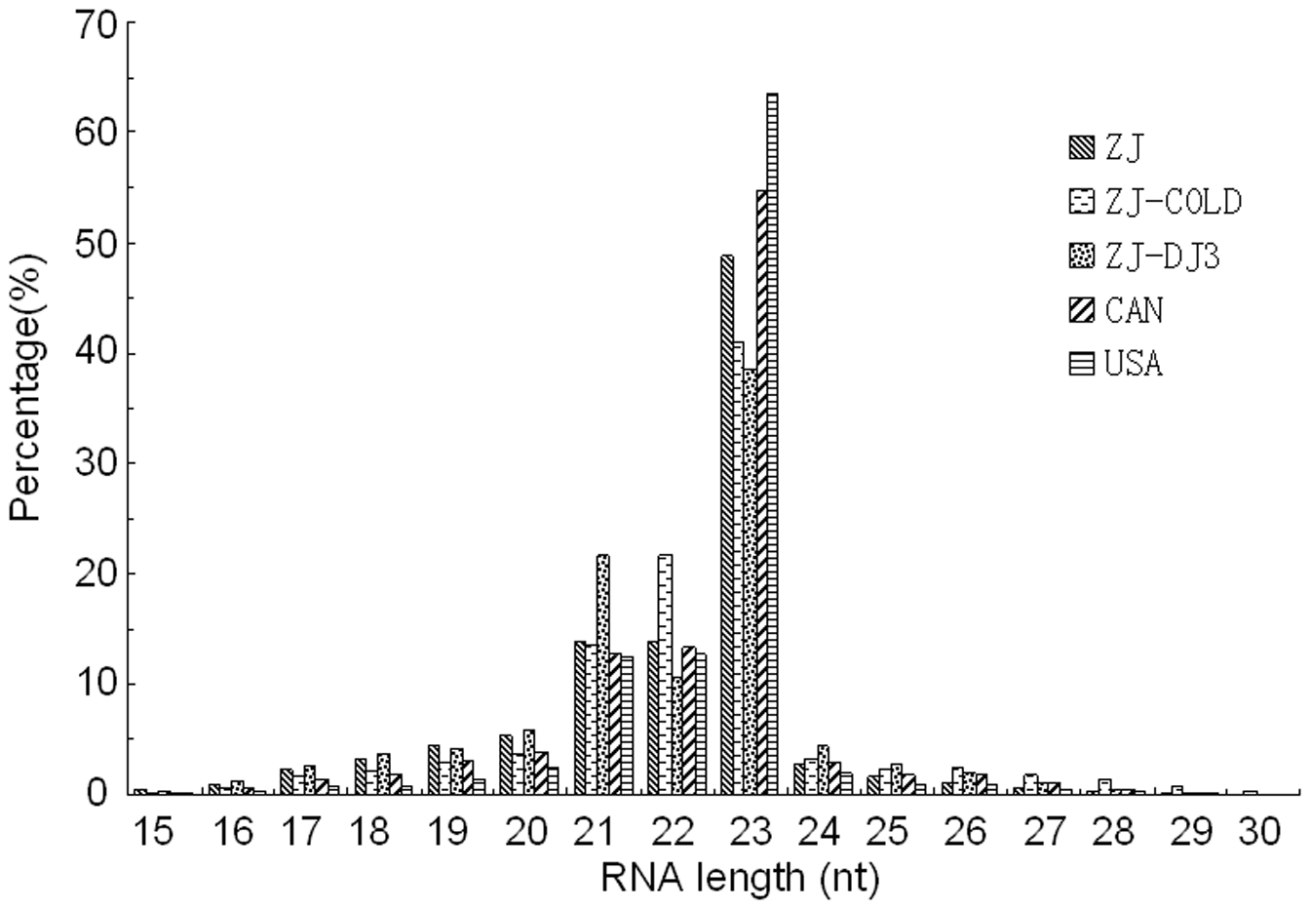
Length distribution of sequenced small RNAs.

Five different small RNA libraries were constructed and sequenced using Illumina/Solexa sequencing technology (Table 1), including one invasive sample (ZJ, ‘R’ form, from Zhejiang, China), two native samples (CAN, ‘M’ form, from Canada, detail unknown; and USA, ‘R’ form, from Texas, USA), one cold stressed sample (ZJ-COLD, ‘R’ form, from Zhejiang, China) and one dispersal J3 stage sample (ZJ-DJ3, ‘R’ form, from Zhejiang, China). Except for ZJ-DJ3, all samples were sequenced using full stages (including eggs, larvae and adults) in propagation cycles. A total of ∼753Mb small RNA data (∼103.24–174.63Mb per library) was produced. Sequencing details are listed in Table 1. Except for the cold stressed library (ZJ-COLD), the sequenced reads of each population are approximately the same (∼8,500,000 reads). The length distributions of small RNAs sequenced in the five libraries were similar (Figure 1). Small RNAs of 21–23nt in length were the most abundant.

**Table 1:** Summary of the deep sequencing results in five small RNA libraries of *B. xylophilus.*

Library name	Nematode Source	Description	No. of sequenced reads	No. of unique reads	No. of clusters
ZJ	Zhejiang, China	All nematode stages in propagation cycle, ‘R’ form	8,481,706	476,094	271,852
ZJ-COLD	Zhejiang, China	All nematode stages in propagation cycle, ‘R’ form, cold shocked at 4°C	5,061,769	264,782	177,237
ZJ-DJ3	Zhejinag, China	3rd juvenile stage (J3) in dispersal cycle, ‘R’ form	8,793,150	885,411	534,187
CAN	Canada (detail unknown)	All nematode stages in propagation cycle, ‘M’ form	8,452,428	468,652	328,123
USA	Texas, USA	All nematode stages in propagation cycle, ‘R’ form	8,496,594	307,140	206,042

**Figure 2:**
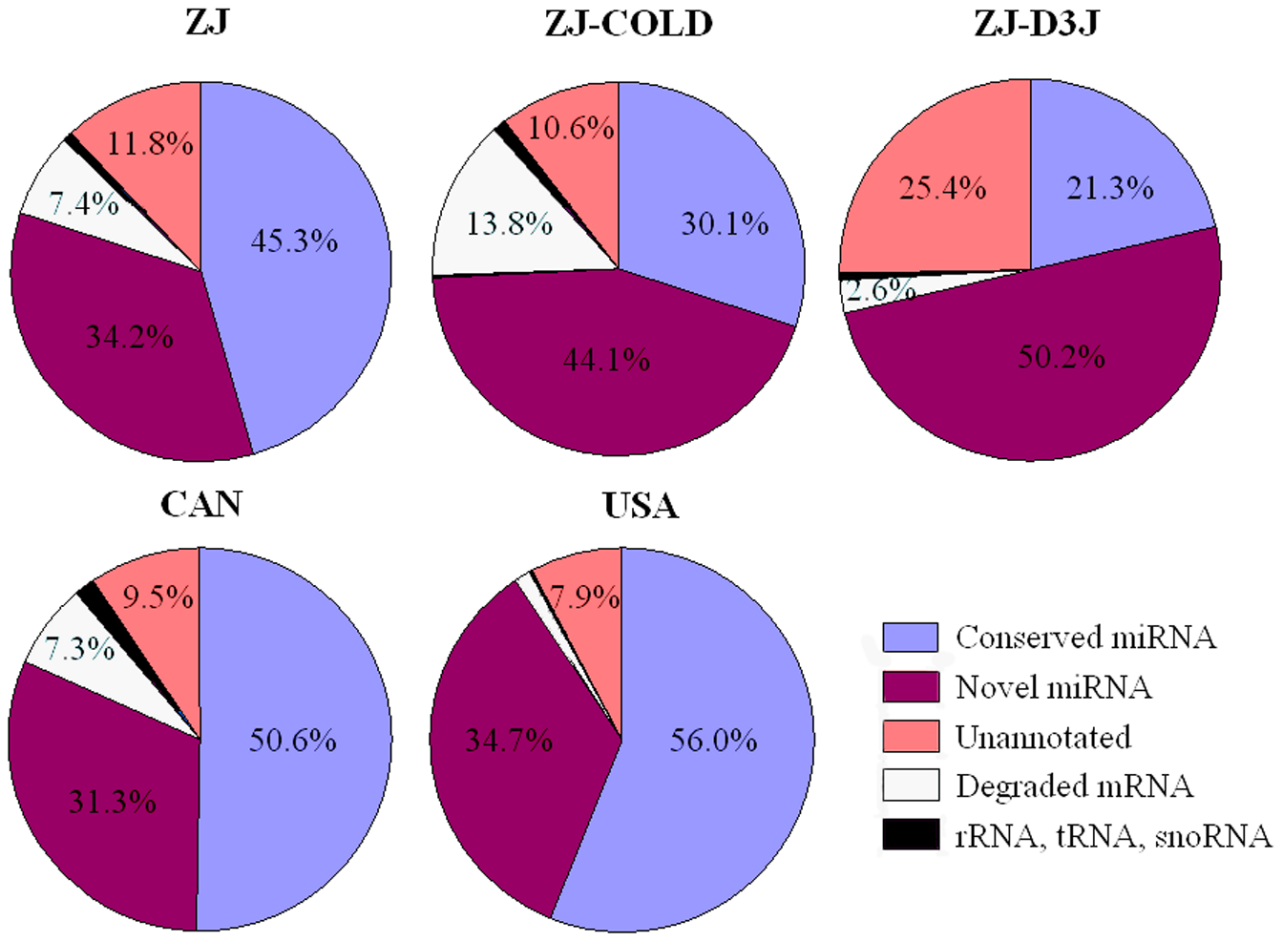
Distribution of different small RNA classes.

After removing the low-quality reads, sequence reads were converted into unique sequence tags with associated counts. Identical sequence reads were further grouped into clusters based on their sequence similarity. It was shown that the dispersal J3 small RNA library (ZJ-DJ3) had the highest number of unique sequence tags and sequence clusters, whereas ZJ-COLD had the least (Table 1). By MegaBLAST searching, sequenced reads that perfectly matched ESTs of *B. xylophilus* and *B. mucronatus*, and with known rRNAs of nematodes, were removed. The remaining clean sequenced reads were used to search for both conserved and novel miRNAs. It was shown that miRNAs were the most abundant small RNA class in all of the five small RNA libraries, consisting of more than 70% (71.5%–90.7%) of sequenced small RNAs in each library (Figure 2).

### Identification of MicroRNAs from the small RNA libraries by computational algorithms

By a computational pipeline, a total of 810 miRNA candidates were predicted from the five small RNA libraries of *B. xylophilus* and 59% of them were detected in all libraries. Of these, 49 conserved miRNA candidates comprised of 30 miRNA families were found based on the consensus ‘seed’ region (2–7nt in 5′ end of mature miRNA) with the published miRNAs (supplemental Table S1). Nineteen miRNAs were homologues with known *C. elegans* miRNAs. Thirty one miRNA candidates were detected in all the five libraries, in which, *miR-100*, *miR-1*, *let-7*, *miR-72*, *miR-71*, *miR-34* and *miR-252* were abundantly expressed (Table 2). The sequenced counts of these miRNAs could be as high as more than one million, several orders of magnitude greater than most small RNAs detected here. The most abundant miRNA was *miR-100*, with more than 2 million counts reaching an abundance of more than 12,000 copies per Mb in the USA library. However, *miR-10*, *miR-184*, *miR-228*, *miR-275*, *miR-279*, *miR-44* and *miR-81* were detected only in ZJ-DJ3 (supplemental Table S1).

**Table 2:** Sequences, abundance and homologues of top ten predicted conserved miRNA candidates in five small RNA libraries.

Name	Sequence	Counts/Mb					Homologues*
		ZJ	ZJ-COLD	ZJ-DJ3	CAN	USA	
*miR-100*	AACCCGUAGAUCCGAACUUGUGU	9,798.0	234.8	150.0	9,283.8	12,227.3	*hsa-miR-100*
*miR-1*	UGGAAUGUAAAGAAGUAUGUAG	1,133.8	3,823.0	587.9	1,342.1	1,052.7	*cel-miR-1*
*let-7*	UGAGGUAGUAGGUUGUAUAGUU	360.2	1,307.8	507.0	302.9	482.1	*cel-let-7*
*miR-71*	UGAAAGACAUGGGUAGUGA	79.9	21.7	63.0	86.3	87.3	*cel-miR-71*
*miR-34*	UGGCAGUGUGGUUAGCUGGUUG	62.2	84.2	248.7	49.7	42.7	*cel-miR-34*
*miR-72*	AGGCAAGAUGUUGGCAUAGCUGA	49.0	270.8	271.5	67.9	60.4	*cel-miR-72*
*miR-252*	CUAAGUAGUAGUGCCGCAGGUAA	30.7	58.3	16.3	29.7	40.4	*cel-miR-252*
*miR-87*	GUGAGCAAAGUUUCAGGUGUGC	7.3	8.5	6.5	10.6	4.1	*cel-miR-87*
*miR-86*	UAAGUGAAUACUUUGCCACAGUC	3.8	4.9	1.2	4.4	4.7	*cel-miR-86*
*miR-50*	UGAUAUGUCUAGUAUUCUUGGG	3.8	1.6	3.0	5.9	4.8	*cel-miR-50*

Note: * Homologues are identified by homology search in miRBase (release 14.0). *cel*, *C. elegans*; *hsa*, *H. sapiens.*

For novel miRNAs, although there are different computational methods to be developed for miRNAs prediction from deep-sequencing small RNA data, most need additional genome sequences as background data. As the genome data of *B. xylophilus* is unpublished at present, we developed a computational pipeline to predict novel miRNA/miRNA* candidates following Wei's method [30], which is based on a 1–2nt 3′ overhang pattern generated by Dicer cleavage during mature miRNA generation. A total of 761 novel miRNA duplex-like pairs were predicted (supplemental Table S2). Among these, 447 were found in all libraries. The top ten of the most abundant sequenced candidates are listed in Table 3. However, 17.3% of novel miRNA candidates were only detected in one library.

**Table 3:** Sequences and abundance of top ten predicted novel miRNA candidates in five small RNA libraries.

Name	Sequence	Counts/Mb					Corresponding miRNA name
		ZJ	ZJ-COLD	ZJ-DJ3	CAN	USA	
*Candi-1*	UGAGAUCAAAGGUUUUAGGGUAU	5,238.1	11,375.5	8,093.8	6,406.6	7,766.1	*bxy-novel-18*
*Candi-2*	UGACUAGAUCCAUACUCAGCU	2,216.1	1,843.6	5,488.6	1,859.0	2,535.7	*bxy-miR-45*
*Candi-3*	AACCCGUAGAAUUUACUUUCGUU	722.2	76.4	368.8	853.4	676.0	*bxy-novel-02*
*Candi-4*	UGAGGUAUUGUCAUCAUGUCUAU	433.9	550.4	291.4	455.7	509.3	*bxy-novel-11*
*Candi-5*	UGAGAUCAGACUAGACUCAUCU	426.8	160.7	111.4	412.7	407.6	*bxy-novel-13*
*Candi-6*	UGAGAUCAUAGAUUUAGGGUAC	206.3	392.4	115.8	241.9	272.6	*bxy-novel-07*
*Candi-7*	UGAGAUCAUAGAUUUAGGGUA	177.2	434.3	58.9	201.6	218.1	--
*Candi-8*	UCCCUGAGACUAUAACUGU	172.0	128.8	10.1	179.8	141.2	--
*Candi-9*	UCCCUGAGACUAUAACUGUGA	148.3	75.0	113.5	166.8	135.1	--
*Candi-10*	UGAGAUCGGUUCGGAUUCGUCA	136.2	656.0	93.4	122.0	202.0	--

**Figure 3:**
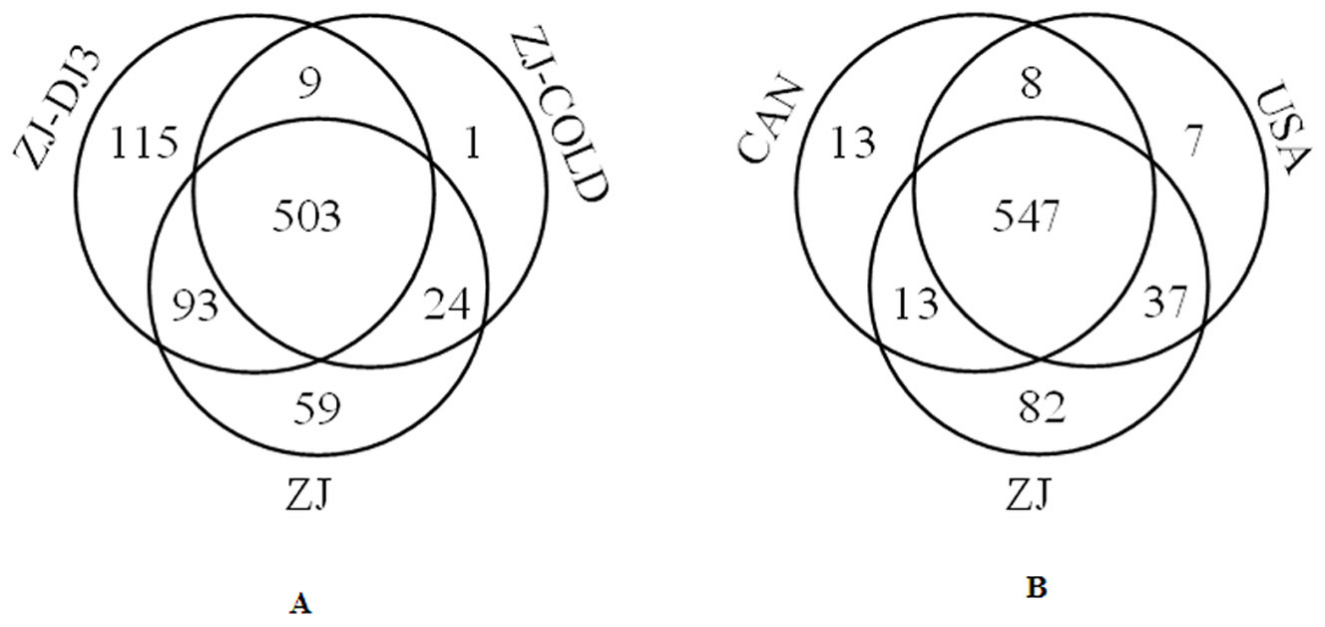
Venn diagrams for the number of predicted miRNAs in different conservation groups. (A) 804 miRNA candidates in three specific phases libraries (normal, cold-shocked and dispersal) of the Chinese population. (B) 707 miRNA candidates in three geographic population libraries (Chinese, American and Canadian).

Comparing the abundance of miRNAs in these five small libraries, it was shown that ZJ-DJ3 was enriched with the most miRNA candidates (43 conserved and 677 novel), and ZJ-COLD with the least (38 conserved and 499 novel). As shown in Figure 3, among the three special-phase libraries of the Chinese population (ZJ, ZJ-COLD, ZJ-DJ3), a lot of special miRNAs (115) belonged to the dispersal stage (ZJ-DJ3). While, among the three geographic population libraries (China, Canada and USA), more of the special miRNAs (82) belonged to the Chinese library (ZJ).

In addition, we have obtained a small dataset of pinewood nematode genome sequences by using the 454/Roche sequencing approach (data unpublished). When we combined this dataset with the small RNA libraries, we identified five additional novel miRNAs and their corresponding pre-miRNAs (*bxy-novel-10/17/21/26/29*, see in supplemental Table S3) by the use of miRDeep [31], despite these very low expression abundances in general.

### Experimental verification of miRNAs

**Figure 4:**
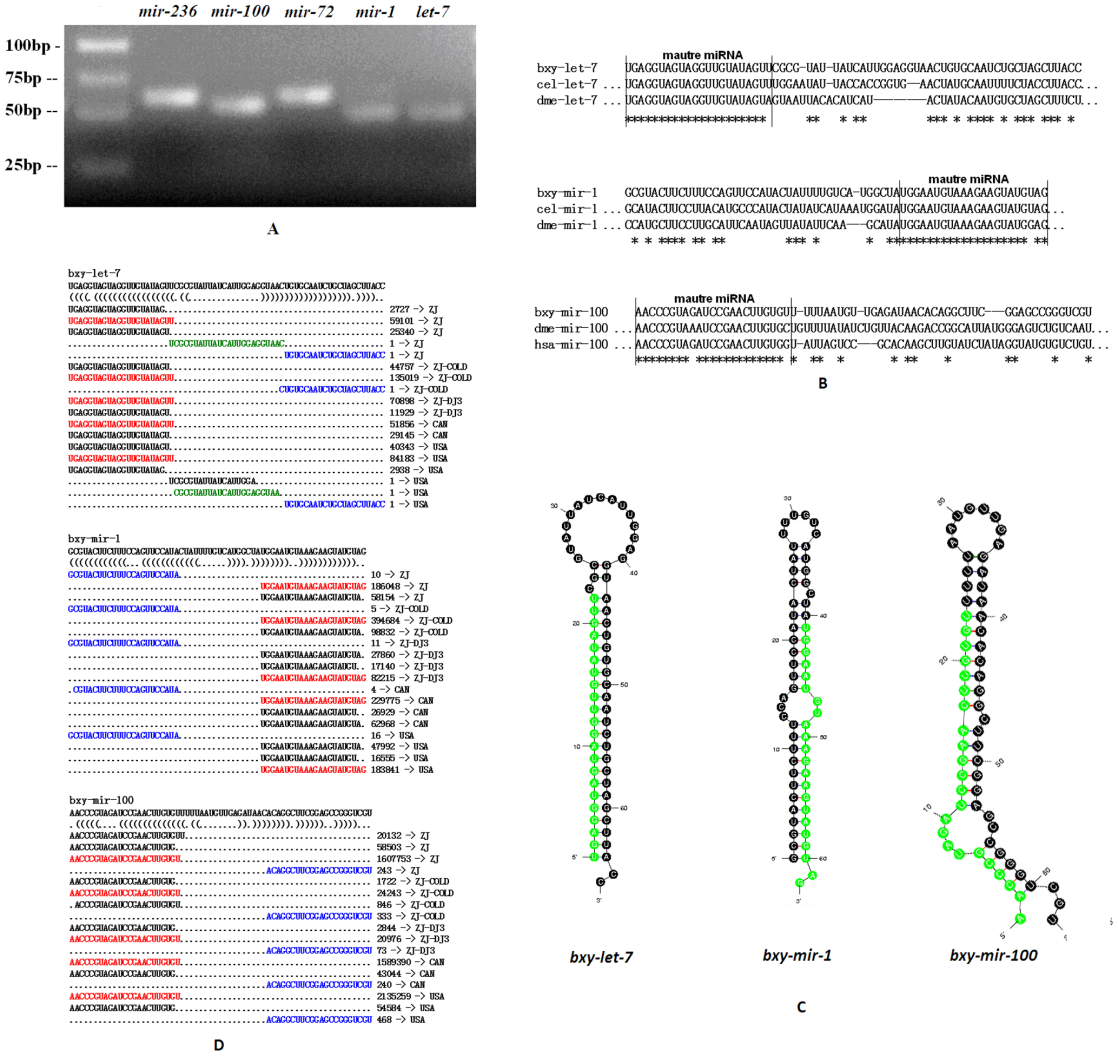
Conserved miRNAs verified in *B. xylophilus*. (A) Electrophoretic analysis of conserved miRNA precursor PCR products. (B) Sequence alignment of *B. xylophilus* miRNAs with known animal miRNAs. (C) Hairpin structures of *B. xylophilus let-7*, *mir-1* and *mir-100* miRNA precursors. Nucleotide bases of mature miRNAs are highlighted with green color. (D) Small RNAs derived from the conserved miRNA precursors. The sequence of the *let-7*, *mir-1* and *mir-100* hairpins depicted above their bracket-notation secondary structure as determined by RNAfold (Hofacker *et al*., 2003). Below, each of the small RNA sequences that matched the hairpins is listed, with the number of reads representing each sequence and the corresponding small RNA libraries are shown in the right side. The dominant miRNA sequence is red; the dominant miRNA* species is blue; and the loop-containing sequence is green.

The predicted miRNA/miRNA* duplexes were verified by amplification of miRNA precursors using polymerase chain reaction (PCR). Twenty conserved miRNA precursors encoding 22 mature miRNAs were detected by PCR (supplemental Table S3, Figure 4A). Clustal W alignments with published miRNA precursors in miRBase highlight the fact that these *B. xylophilus* miRNA precursors are conserved with miRNAs precursors in *C. elegans*, flies and human (Figure 4B). Thus, we named these miRNAs after their homologues. Among these conserved miRNAs, *miR-100*, *miR-45*, *miR-1*, *miR-72* and *let-7* were sequenced most abundantly. The sequences of these pre-miRNAs and their miRNA homologues in other animals are listed in supplemental Table S3.

All of these miRNAs can be folded into characteristic miRNA stem-loop secondary hairpin structures (1-2nt 3′ overhang, etc.) (Figure 4C, see supplemental Figure S1 for all). Alignments of sequenced miRNAs and their precursor sequences provide a vivid view of mature miRNAs processed by Dicer. Figure 4D shows the different expression patterns of several miRNAs in each of the sequenced small RNA libraries (Figure 4D, see supplemental Figure S2 for all), which provide strong evidences to support the identity of these conserved miRNAs in *B. xylophilus*.

Interestingly, two miRNAs* are also frequently sequenced: miR-234* and miR-9*. Neither of them have potential homologues with known metazoan mature miRNAs. However, miR-9* shares the same ‘seed’ with *hsa-miR-320a/b/c/d*, while miR-234* might be a *B. xylophilus* species-specific miRNA (Table 4).

**Table 4:** List of ten species-specific miRNAs of *B. xylophilus.*

Seed	Name	Sequence	Counts/Mb					Homologue(s)
			ZJ	ZJ-COLD	ZJ-DJ3	CAN	USA	
GGGUAU	*bxy-miR-234*	UGGGUAUUCUCUGGCAAUGGACA	1.8	0.8	0.6	1.1	1.0	ND
AUAUCU	*bxy-novel-06*	AAUAUCUGAAAAGUUGGUGUG	118.0	121.0	16.3	112.9	86.0	ND
GCACUC	*bxy-novel-08*	AGCACUCGACGUAUGAAAUCGUUU	2.2	2.8	1.2	4.9	3.1	ND
UUGCGA	*bxy-novel-09*	UUUGCGACUGUUUUCAGGCCUUU	87.5	704.8	86.2	64.1	56.0	ND
CCAGUU	*bxy-novel-10*	UCCAGUUCGAGAUGACGCCU	0.8	0.3	0.0	0.0	3.2	ND
GGGGCG	*bxy-novel-21*	GGGGGCGAAAUAGGAUCGACA	1.0	0.0	0.0	0.3	0.8	ND
AUAGGA	*bxy-novel-23*	UAUAGGAAAUGCGUCACAAGCGAU	11.8	25.4	1.7	6.9	8.1	ND
AAAAUG	*bxy-novel-28*	UAAAAUGGCUGUCAGGUGUAAU	3.7	0.0	7.0	5.2	6.2	ND
ACUGGU	*bxy-novel-30*	AACUGGUCGUCAAAAUCAAAAG	1.2	0.7	0.1	0.0	6.7	ND
UUUCAU	*bxy-novel-32*	UUUUCAUGCCUUUGUAUUCAUA	0.9	0.9	0.0	0.5	0.6	ND

ND: not detected.

**Figure 5:**
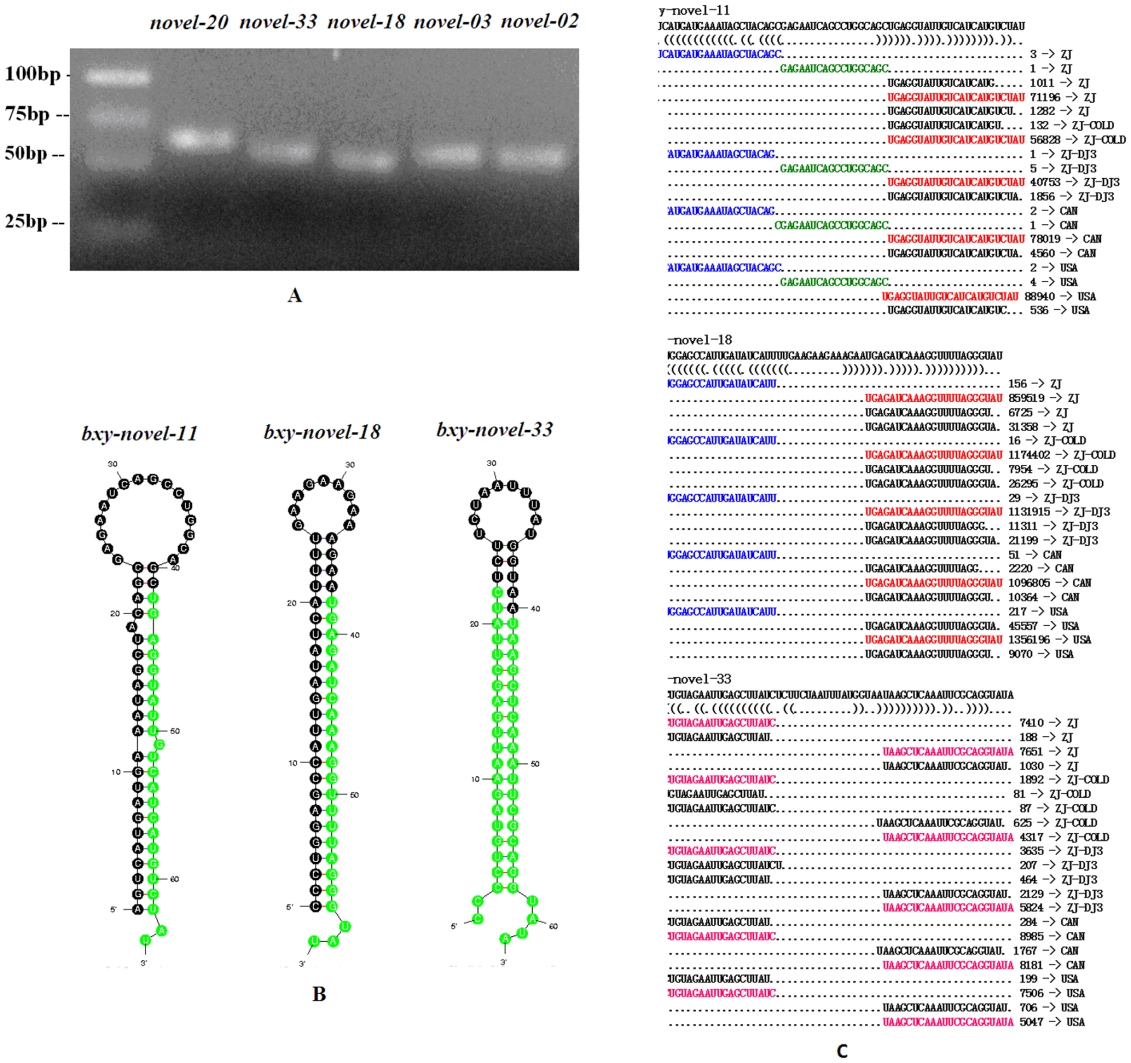
Novel miRNAs detected in *B. xylophilus*. (A) Electrophoretic analysis of novel miRNA precursor PCR products. (B) Hairpin structures of pine wood nematode *novel-11*, *novel-18* and *novel-33* miRNA precursors. Nucleotide bases of mature miRNAs are highlighted with green color. (C) Small RNAs derived from the novel miRNA precursors. The sequence of the *novel-11*, *novel-18* and *novel-33* hairpins depicted above their bracket-notation secondary structure as determined by RNAfold (Hofacker et al., 1994). Below, each of the small RNA sequences that matched the hairpins is listed, with the number of reads representing each sequence and the corresponding small RNA libraries are shown in the right side. The dominant miRNA sequence is red; the dominant miRNA* species is blue; and the loop-containing sequence is green. For *novel-33*, the equally expressed two miRNAs (*novel-33-5p* and *novel-33-3p*) are shown in pink color.

Thirty three novel miRNA precursors encoding 35 mature miRNAs are verified by PCR (Figure 5A). A homology search in miRBase revealed no known miRNA precursors. Thus, we classified these miRNAs and precursors as novel *B. xylophilus* miRNAs and named them as ‘novel’. All of the novel pre-miRNAs have secondary structures of characteristic stem-loop hairpins (Figure 5B, see supplemental Figure S1 for all) and their alignments with sequenced small RNAs further support the identification of their identities of miRNA precursors (Figure 5C, see supplemental Figure S2 for all). Among them, 28 novel miRNAs were detected in the small RNA libraries (supplemental Table S3).

By BLAST search in miRBase, no homologues of the 35 mature miRNAs were found. However, 26 novel miRNAs showed a conserved resemblance to known metazoan miRNAs, including 19 miRNAs of *C. elegans* and 15 miRNAs of *Drosophila melanogaster* (supplemental Table S3). For example, *novel-07*, *novel-13* and *novel-18* share the ‘GAGAUC’ motif with *cel-miR-1834* and *dme-bantam*. We suggest that these miRNAs can be classified into the same miRNA group, even though the sequences at flanking sites of the conserved region are always diverged.

Nine miRNAs did not appear to share conserved sequence with known miRNAs, and can be classified as species-specific miRNAs in *B. xylophilus* (Table 4). We further used these miRNAs plus miR-234* encoded by *bxy-mir-234* to BLAST search in the GeneBank nucleotide database to check whether any of them could have potential homologues in any species. We found no potential homologues.

### Expression profiles of authentic miRNAs in the five small RNA libraries

**Figure 6:**
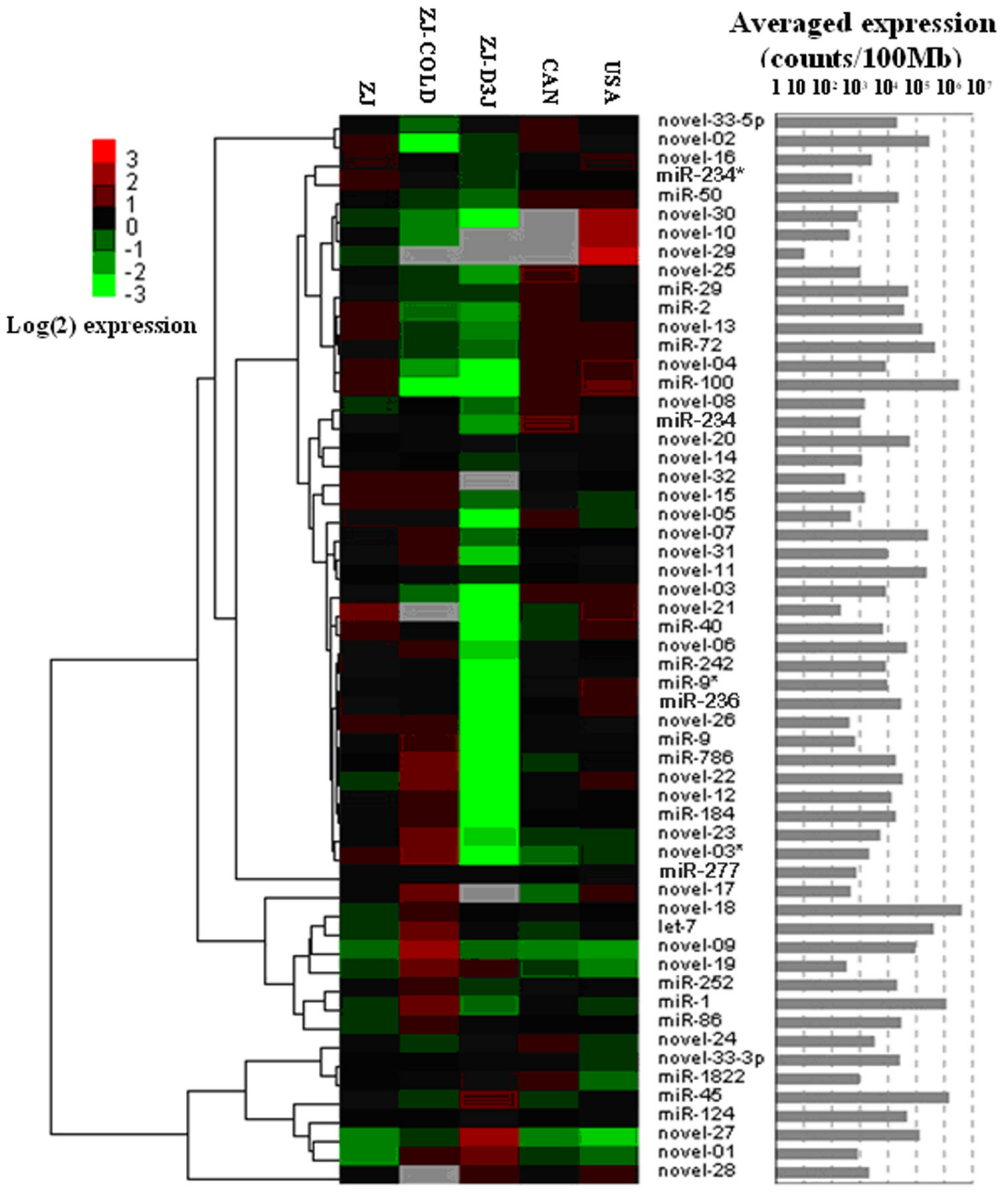
Heat map of the expression profiles of 57 verified miRNAs in different small RNA libraries.

The expression profiles of the verified 57 miRNAs in different RNA libraries are shown in Figure 6. Fifty miRNAs were observed in all the small RNA libraries, while 7 miRNAs at very low expression level (e.g. *novel-29*) were not detected in at least one library. In general, more than half of the miRNAs had an abundance of less than 50 counts/Mb. The average expressed abundances of miRNAs in each small RNA library differ: the most abundant were in the USA library (∼31,000 counts/Mb), and the least abundant were in the ZJ-DJ3 library (∼19,000 counts/Mb). In contrast to our finding that miRNAs in the three libraries (ZJ, CAN and USA) showed a similar abundance (Fig. 6), the miRNAs with different expression patterns were mostly enriched in the ZJ-COLD and ZJ-DJ3 libraries.

Approximately 80% of the miRNAs in the libraries of the propagative (ZJ) and the dispersal (ZJ-DJ3) stages (supplemental Table S3 and Fig. 6) had very different expression levels. Most of them were down-regulated in the dispersal stage, such as *miR-100*, *miR-1*, *miR-72*, *novel-02*, *novel-13*, *novel-07* and *novel-06*. In the ZJ-DJ3 library these were obviously less expressed than in ZJ library. In contrast, *miR-45*, *novel-27* and *miR-124* were more frequently sequenced in the ZJ-DJ3 library.

Obvious variations in miRNAs abundance were found between the normal temperature library (ZJ) and the cold-shocked library (ZJ-COLD). *Novel-18*, *miR-1*, *let-7*, *novel-09* and *novel-07* were much more abundant in the ZJ-COLD library; while, *miR-100*, *novel-02*, *miR-72* and *novel-13* were more abundantly expressed in the ZJ library (supplemental Table S3 and Fig. 6).

The differences in expression levels were not prominent among the three libraries from the different geographic populations (ZJ, USA, CAN) and no negative correlations of miRNA expression patterns were observed among these libraries.

### Other small RNAs

**Figure 7:**
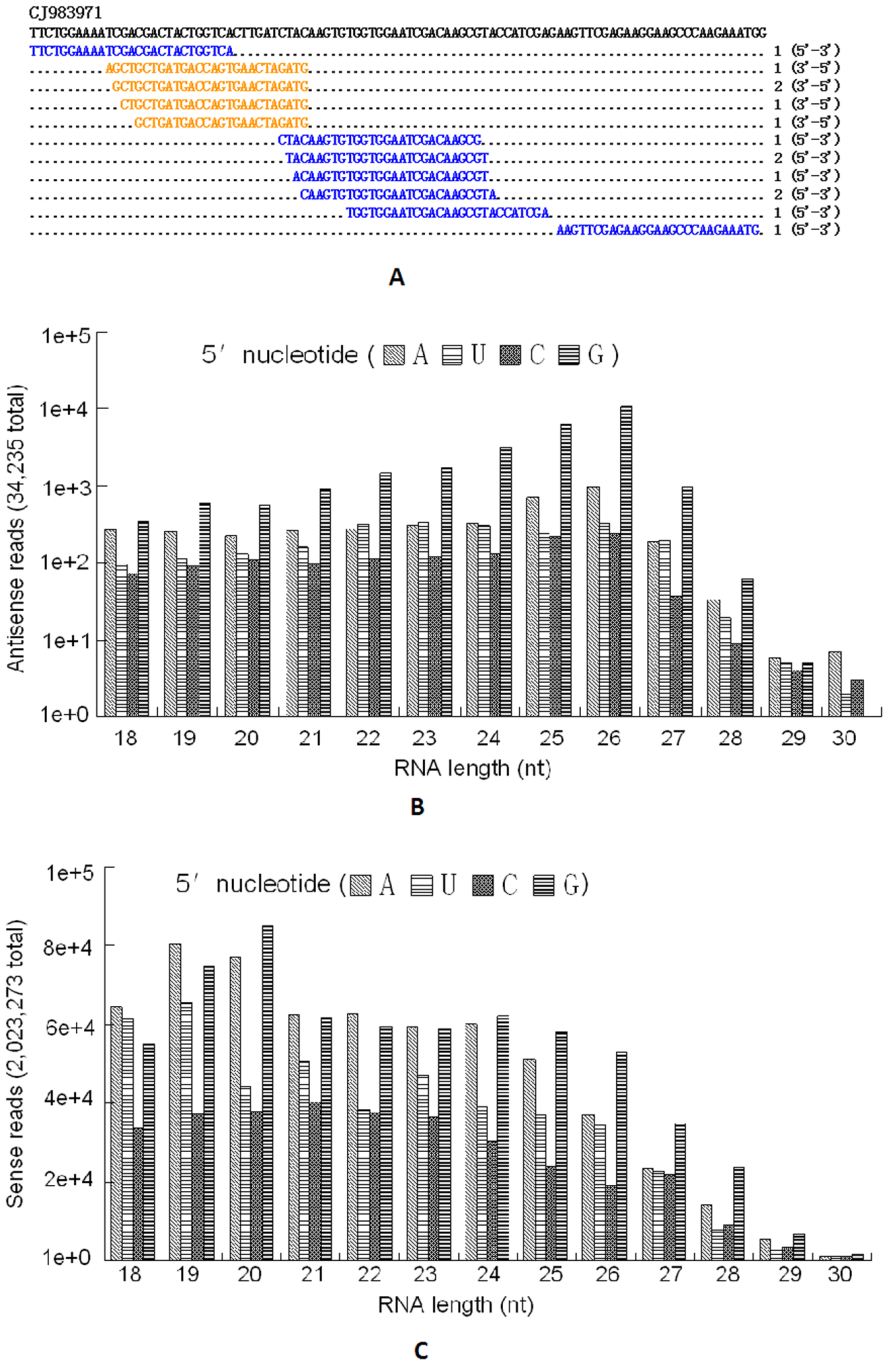
Small RNA reads match to *B. xylophilus* mRNA in all libraries. (A) Portions of an EST aligned with small RNA reads that matched the sense (blue) and antisense (orange) strands. (B) The length and initial nucleotide distribution of the antisense reads. (C) The length and initial nucleotide distribution of the sense reads.

Besides miRNAs, sequences which matched the sense and antisense strands of ESTs of *B. xylophilus* were identified as endo-siRNAs. An example is shown in Figure 7A. In general, these endo-siRNAs have a 5′-G characteristic and are enriched with reads of ∼26nt in antisense strands (Figure 7B) and 20nt in sense strands (Figure 7C). However, endo-siRNAs in all the five small RNA libraries were less frequent. In all small RNA libraries, only 34,235 (less than 0.1% of total) reads were recognized as endo-siRNAs and 2,023,273 reads (5.15% of total) were identified to be degraded mRNA fragments. The rest of the sequences in the small RNA libraries, however, were not identified. Reasons included the limitation of read length and the lack of background data (e.g. transposon sequences), and many small RNA species (e.g. Piwi-interacting RNA). Therefore, some of the sequenced data still remains unclassified (∼14% of total sequenced reads) (Figure 2).

### Prediction of miRNA targets

Although miRNAs usually function on their target genes by binding to sequences in the 3′ untranslated regions (UTRs), it is documented that they can also regulate the expression of their target genes by binding in protein coding regions [2]. Since little information about *B. xylophilus* 3′ UTR is available, 13,340 *B. xylophilus* ESTs were used for prediction of miRNA targets by using the program miRanda. Only ESTs that were potential homologues of *C. elegans* and had a miRanda score of 150 or above were considered. In total, 2,151 ESTs were predicted to contain at least one target site of the 57 mature miRNAs identified in our study (supplemental Table S4). Most of the miRNAs had multiple target sites (e.g. *bxy-let-7*), suggesting that these miRNAs are functionally divergent. Similarly, a single EST could be potentially targeted using several miRNAs (e.g. CJ979180). According to the annotation of homologous *C. elegans* genes in WormBase (www.wormbase.org), these targeted ESTs can be grouped into 11 molecular function categories (Table 5). The majority of EST targets fall into the categories of binding, catalytic activity, transporter activity and structural molecular activity.

**Table 5:** Predicted target functions of the identified miRNAs of *B. xylophilus.*

Molecular function	No. of ESTs
Binding	969
Catalytic activity	886
Structural molecule activity	223
Transporter activity	199
Enzyme regulator activity	81
Transcription regulator activity	33
Translation regulator activity	25
Electron carrier activity	21
Molecular transducer activity	18
Antioxidant activity	11
Proteasome regulator activity	2

A higher miRanda score might give us a more reliable miRNA target predicted result. The highest score for predicted targets is for CJ985685, best annotated as Cell-death-Related Nuclease (*crn-1*) in WormBase by a homology search. This EST is a potential target of both *bxy-novel-18* and *bxy-novel-07* (supplemental Table S4). The two miRNAs are significantly highly expressed in the cold shocked small RNA library, ZJ-COLD (Figure 6). Although we could not find published experimental evidence of miRNAs targeting *crn-1* in *C. elegans* and other animals, our finding here might provide some clues for further identification of the miRNA targets.

A total of 74 records of complete or partial coding regions of *B. xylophilus* genes are published in GeneBank thus far. We derived a 3′ UTR dataset based on the GeneBank description and used miRanda to scan for potential miRNA target sites. Only 43 miRNA target sites of 19 miRNAs were predicted that corresponded to 12 *B. xylophilus* genes using a miRanda score of 50 (Table 6, supplemental Table S5). Thirty six target sites belong to FMRF amide-like peptide gene families, including *flp-2*, *flp-3*, *flp-6a/b/c/d* and *flp-14*.

**Table 6:** Predicted miRNA targets of known *B. xylophilus* genes.

*B. xylophilus* Gene	GenBank Accession No.	3′ UTR Targeting miRNA	No. of target sites
Actin	EU100952	*let-7*	1
*β-1,4*-endoglucanase	AB179544	*miR-86*	1
*flp-14*	EF622046	*miR-184*	1
*flp-2*	EU930826	*novel-26*	2
*flp-3*	EF422867	*miR-40/72/184, novel-08/10/17/26/27*	9
*flp-16a*	FJ151415	*miR-124/184/242, novel-29*	4
*flp-16b*	FJ151416	*miR-184/242, novel-16/17/21/29*	7
*flp-16c*	FJ151417	*miR-50/184/242, novel-27/29*	5
*flp-16d*	FJ151418	*miR-242, novel-16/17/21/27/29*	7
*hsp-90*	EF490991	*novel-21*	1
HDAC	FJ423634	miR-234*	1
UBC3	EU333281	*novel-13/20/26/27*	4

Note: *flp*, FMRF amide-like peptide; *hsp*, heat shock protein; HDAC, histone deacetylase protein; UBC3, ubiquitin conjugating-3 enzyme.

## Discussion

### Efficiency of miRNA prediction by the computational pipeline

As the whole genome sequences of *B. xylophilus* are unpublished at present, it was therefore a challenge to discover novel miRNAs in *B. xylophilus*. Normally, Illumina/Solexa sequencing could only produce reads of no more than 30nt in length in the small RNA libraries we constructed. Such reads are not long enough to depict miRNA precursors, which are usually longer than 50nt. Therefore, many of the methods utilized in searching for novel miRNAs, including mapping to genome, scanning potential stem-loop hairpins by computational methods etc., were not applicable to our study. Wei *et al.* [30] introduced a new method to discover novel miRNA precursors in migratory locust, *Locusta migratoria*. This method is based on a 1-2nt 3′-overhang pattern of a miRNA/miRNA* duplex, which was processed by Dicer cleavage during the miRNAs maturation process. With their method, we further developed a computational pipeline to predict novel miRNA/miRNA* candidates in *B. xylophilus* (see Materials and Methods).

To verify the authenticity of predicted miRNA candidates used with a dataset from the 454 Life Sciences/Roche sequencing approach (data unpublished), we first predicted miRNA precursors by electronic PCR (e-PCR), and then further identified them by experimental PCR. We assumed that miRNA precursors must have characteristic miRNA stem-loop structures. However, only 53 miRNA precursors coding 57 miRNAs were obtained. Precursors for a lot of miRNA candidates have not yet been amplified. Two things might lead to this result: a false positive rate or an imperfect 454 dataset. With respect to the false positive rate, Wei and his associates assessed that it would be lower than 40% [30]. To test the efficiency of this method, we first used the published mature miRNAs and their corresponding miRNAs* in miRBase, the data used by Ruby *et al.* [15] and Wei *et al.* [30], to construct a reference small RNA library. A true positive rate around 0.5 was observed. The false positive rate might relate to the dataset size, and increase as data increased. Because our dataset for miRNA prediction is much larger in this study, this might lead to a much higher false positive rate. Nevertheless, the method is feasible for predicting miRNA/miRNA* when no genome sequence is available. In this study our 454 dataset is small and imperfect. Many miRNAs cannot be matched to the dataset. In the future, when the genome sequence is available, we can then obtain a comprehensive understanding of *B. xylophilus* miRNAs and targets.

### Characteristics of *B. xylophilus* miRNAs

In this study, 57 authentic miRNAs (20 conserved and 37 novel) were discovered in *B. xylophilus* by computational and experimental methods. It is the first description of miRNAs in plant parasitic nematodes. We found that many *B. xylophilus* miRNAs are conserved with metazoan miRNAs in the current miRBase release. We can group all of the 57 *B. xylophilus* miRNAs into 47 miRNA families by ‘seed’ conservation (supplemental Table S6). Analysis of the evolutionary conservation of these miRNAs with previously known miRNAs belonging to *C. elegans*, flies and human revealed that 13 miRNA families (including 18 miRNAs) are conserved in the evolution of animals. 38 miRNAs are conserved with *C. elegans* in the ‘seed’ region and can be divided into 27 groups (supplemental Table S6), 23 of which can be sorted into 14 different *C. elegans* family groups based on sequence identity at the 5′ end [32].

Interestingly, three miRNAs (novel-33-5p, miR-184 and miR-9*) are conserved with fly and human miRNAs in the seed region, but have no corresponding family members in *C. elegans* and two other evolutionary closely-related parasitic nematodes *Bugia malayi* [18] and *Pristionchus pacificus* [17] (supplemental Table S6). For example, *miR-184* is found in many animals from the acorn worm to primates in the miRBase, suggesting that it fulfills an essential function. Iovino *et al*. [33] reported that *miR-184* has multiple roles in the *Drosophila* female germline development. We don't know whether *miR-184* and the other two miRNAs were lost in the nematode lineage evolution, or whether they were not found because of their comparatively lower expression levels, as is true for the deep-sequencing results of *B. xylophilus* (see supplemental Table S3). Identification of these miRNA targets in *B. xylophilus*, in order to determine whether they are conserved in *C. elegans* and other nematodes and knowledge of how they are regulated by miRNAs, may provide some insight into the evolution of parasitism of nematodes.

In our study we found 10 novel *B. xylophilus* miRNAs that are species-specific (Table 4, supplemental Table S3). As in previous studies with four other nematode species we found that less-conserved miRNAs were expressed at lower levels [17]. These 10 species-specific miRNAs tend to be expressed at much lower levels than conserved miRNAs (Table 4, Fig. 6). However, two most abundantly expressed species-specific miRNAs (*novel-06* and *novel-09*) have an essential/unique role in gene regulation in this plant parasitic nematode. In a survey of ESTs from 30 nematode species, about 30–50% of genes in each species seemed to be species-specific [34]. Moreover, as illustrated in the root-knot nematode *Meloidogyne incognita*, plant parasitic nematodes might contain a more divergent gene regulatory network due to their peculiar biology that has evolved in adaptation to parasitic life styles [35]. We believe that identification of the target genes of these species-specific miRNAs would help us to better understand the evolution of plant parasitic nematodes.

We detected 3 miRNA star sequences (miR-234*, miR-9* and novel-03*) and two equally expressed miRNAs (*novel-33-3p* and *novel-33-5p*). MiRNA*s may also play subtle functional roles in regulatory activity [36]. *Mir-9* is an ancient miRNA family in animals. Interestingly, unlike other species in which the 5′ arm is dominant, the 3′ arm is dominant (5′/3′ read ratio: 1/15) in *B. xylophilus*. The 5′ arm belongs to the *miR-9* group while the 3′ miR-9* belongs to another group when classified according to seed conservation (supplemental Table S6). This observation supports the arm-switching hypothesis that some biologically functioning miRNA* species have undergone transition to mature sequences [37]. Evidence of the same miRNA evolution pattern has been identified also in other nematode species [17].

### Potential functions of miRNAs in ecological adaptation of *B. xylophilus*

Knowledge of potential targets may be of highest importance in the elucidation of the microRNA regulatory network. Most of the known metazoan miRNAs target 3′ UTR sequences of mRNAs. Unfortunately, the current data for *B. xylophilus* 3′ UTRs is limited. Alternatively, we utilized the published ESTs and a small dataset of *B. xylophilus* 3′ UTRs to predict miRNA targets. 979 target genes were predicted using miRanda software (supplemental Table S4 & S5). Of course, the existence of false positive targets is unavoidable. We are most interested in following two sorts of targets, viz, heat shock protein coding genes relating to stress adaptation; and also the neuropeptide coding genes that relate to message transmission behaviors. Our goal is to understand the roles of miRNAs in regulation of the nematode's ecological adaptation.

**Figure 8:**
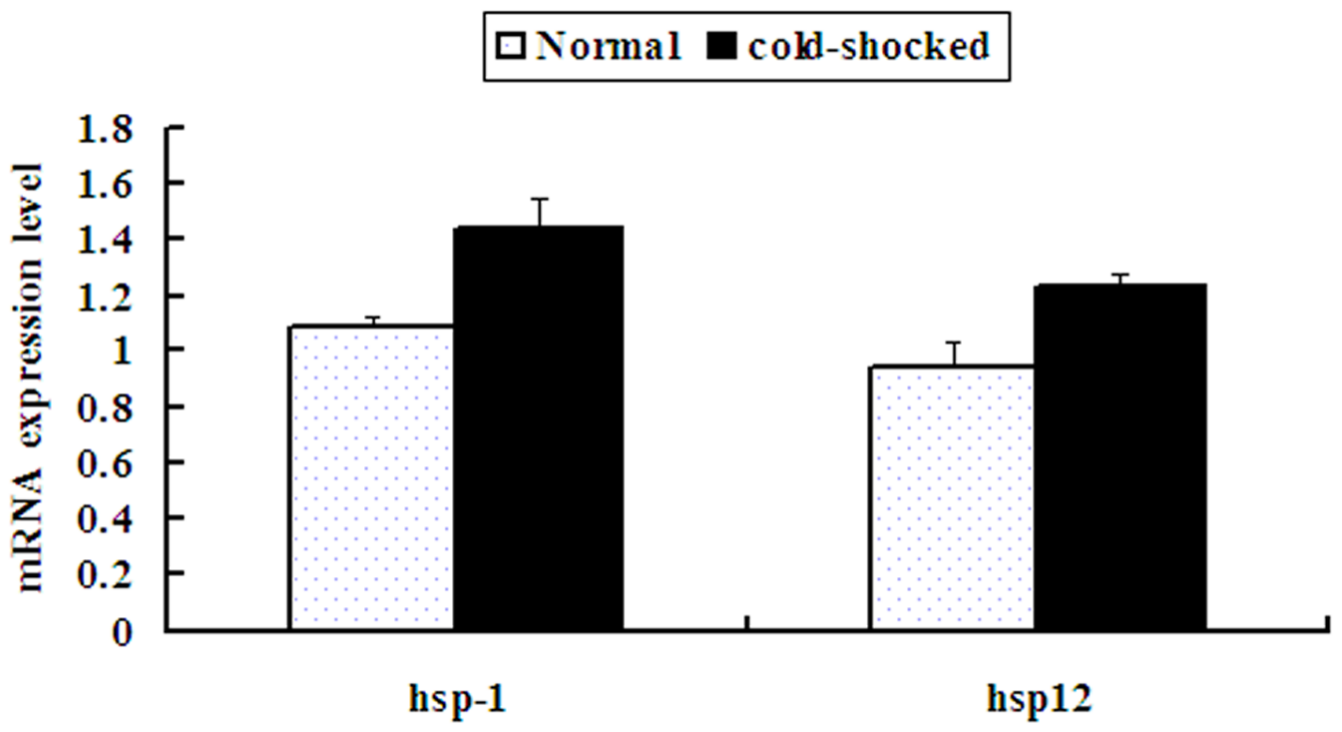
Comparison of mRNA expression levels of heat shock protein genes in *B. xylophilus*. Both genes (*hsp12* and *hsp-1*) have obvious up-regulated expression levels in a cold-shocked status (*P*<0.05).

Heat shock proteins are called stress proteins. Organisms respond to heat or cold shock or other environmental stress by the induction of the synthesis of heat-shock proteins. 72 ESTs identified as heat shock protein coding genes with previously known *B. xylophilus* mRNAs are predicted to have 89 potential target sites of 14 miRNAs (supplemental Table S4). A real-time quantitative polymerase chain reaction (RT-qPCR) was used to quantify mRNA expression levels of heat shock protein genes *hsp12* and *hsp-1* (*hsp-70a*) and to compare the changes of mRNA expression levels in a cold shocked vs. a non-shocked status. As shown in Figure 8, both genes have obvious up-regulated expression levels in a cold-shocked status (*hsp12*: df = 16, t = 2.722, p = 0.15; *hsp-1*, df = 16, t = 3.134, p = 0.006). Two miRNAs (*bxy-novel-10* and *bxy-mir-184*) and four miRNAs (*bxy-novel-07*, *bxy-novel-31*, *bxy-mir-29*, *bxy-mir-40*) have target sites of *hsp12* and *hsp-1* respectively. Although we did not synchronously quantify the expression level of miRNAs with their targets, we referenced the expression levels of these miRNAs in the two libraries (ZJ and ZJ-COLD) and found that *bxy-novel-10*, *bxy-mir-29* and *bxy-mir-40* were down-regulated in the ZJ-COLD library, with *bxy-mir-29* down regulation the more obvious. In addition, *bxy-mir-72* that targeted *hsp-60* was also obviously down-regulated in the ZJ-COLD library. Of course, gene regulation is a complex network and we still know little about how these miRNAs regulate their targets, especially those with no obvious negative correlations to their targets. Except for the human *hsa-miR-320*, which was recently reported to regulate the expression of a heat shock protein, *hsp-20* [38], little information of verified microRNA-regulated *hsp* genes has been reported in animals. Our finding in this study may provide clues for further study of microRNA and heat shock protein targets.

Neuropeptides play important roles in chemical signalling in the central and peripheral nervous systems. FMRFamide-like neuropeptides (FLPs) are the largest family of neuropeptides in nematodes and they modulate sensory and motor functions [39]. In *C. elegans*, FMRFamide-like neuropeptide genes (*flps*) are known to be key genes related to neuronal sensitivity and other behaviors [40]. In our research, seventeen miRNAs were predicted to target the 3′ UTR sequences of six *B. xylophilus* FMRFamide-like neuropeptides genes (*flp*-2/-3/-6/-12/-14/-16) (Table 6, supplemental Table S4 & S5). The expression levels of these miRNAs in the dispersal ZJ-DJ3 library were obviously down-regulated compared with the propagative ZJ library (except *miR-124* and *novel-27*). Although we have not detected the expression level of *flp* genes, we can refer to results of other studies. Kang *et al*. [41] reported that *flp-3* showed 5.1-fold higher expression in the *B. xylophilus* dispersal stage than in the propagative stage. So, the obvious negative correlation (miRNA expression down-regulated and *flp* mRNA up-regulated) clearly indicates that miRNAs participate in the regulation of *flp* genes expression. As we mentioned in the introduction, pinewood nematode has a complex and intriguing life cycle that involves a propagative cycle and a dispersal cycle. The third-stage dispersal juvenile (J3) is a special stage. The juveniles can response to the chemical substances diffused by the *Monochamus* beetle pupa and congregate in the vicinity of the pupal chambers where they molt to the fourth stage dispersal juveniles at the time of the vector beetle eclosion [26], [27]. We postulate that *flp* genes along with their corresponding miRNA regulators take roles in the chemical-mediated interaction that exists between the pinewood nematode and its beetle vectors.

Two well known ancient miRNAs, *let-7* and *miR-1* were much more abundantly expressed in cold shocked nematodes (∼3.4-fold and ∼3-fold more respectively). *Let-7* was discovered in *C. elegans* as a timing regulator controlling the L4-adult transition in development [42], and it is required for regulating normal adult behavior in flies [43]. *MiR-1* was recognized to be a muscle-specific miRNA [44]. It exhibits a striking decline during adult life and it was thought that the drop in *miR-1* levels might promote muscle aging in nematodes [45]. It takes an essential role in maintaining muscle integrity in flies [46]. Considering that nematodes under cold conditions are normally less active, we suggest that the promoted expression levels of *let-7* and *miR-1* in the cold-shocked nematodes might imply enhanced roles for them in regulating genes related to activity. The most abundant novel miRNA, *novel-18*, has its peak expression abundance (11,926 counts/Mb) in the cold-shocked nematodes. The seed region of this miRNA is conserved to *cel-miR-1834/80/81/82* and *dme-bantam* (Table 4). *Cel-miR-80/81/82* are reported to function during adult aging [45], while *dme-bantam* can stimulate cell proliferation and prevents apoptosis in flies [47]. The high expression of *novel-18* in the cold-shocked nematodes may indicate its role in the gene regulatory network and it is worthy of special interest in further research.

Although we tried to find miRNA differences between the ‘R’ form and ‘M’ form (CAN and USA libraries), no remarkable difference existed. We suggest that miRNAs may not be related to the pathogenicity and morphological construction of the female tail. According to the data of computational prediction, more novel miRNA candidates were predicted in the ZJ library, which was collected from an invasive area (supplemental Table S2). The unique miRNA candidates in the ZJ library were more abundant than in the USA native population (82 vs. 7, Fig. 3). Although we do not claim that these miRNA candidates are directly related to the invasiveness of the nematode, at least, the results support the conclusion of other studies showing that the Chinese invasive populations have more abundant genetic diversity than do the native populations [48].

## Materials and Methods

### Nematode sources

Four isolates of the pinewood nematode were used in this study. Among of them, three isolates (each from China, USA and Canada respectively) were cultured on fungal mats of *Botrytis cinerea* grown on 1.5% potato-dextrose agar (PDA) plates at 24–25°C. The Chinese sample was isolated from chips of a dead pine tree of Zhejiang province. The USA sample was isolated from American materials (according to the information of its quarantine number, it was from Texas, USA) intercepted by the Chinese quarantine departments. The Canada sample was donated by Dr. Li (Nanjing Agricultural University, China). For a first culture, the nematode individuals of each sample were isolated from a piece of wood, and after morphological identification under a high-powered microscope, about 100 individuals of each sample were picked out and sterilized with 3% H_2_O_2_ for 10 min. After they were washed four or five times with disinfected distilled water (DDW), the nematodes were cultured on fungal mats grown on PDA plates containing 0.1% Streptomycin Sulfate. Nematodes were isolated with the Baermann funnel method [49] and the nematodes could pass through two or three layers of filter-paper while fungal mats could not. All nematode stages (including egg, larva and adult) in propagative cycles were used for library construction. The Chinese sample was also used for a cold stressed library construction by being cold shocked at 4°C for 48 h before the molecular experiments. The other isolate used for construction of a third-stage dispersal juvenile library was achieved by collecting directly from chips around pupal cells of the vector beetle (*Monochamus alternatus*) in a dead pine tree in winter from the same site where the Chinese propagative isolate was obtained. The sources of nematodes used for this study are listed in Table 1. Fresh cultured or isolated nematodes were washed with 0.1 M NaCl solution and then fully washed several times in disinfected distilled water before the molecular experiments.

### Small RNA library construction and high-throughput sequencing

Five small RNA libraries were constructed (three propagative libraries from different geographic populations, one third-stage dispersal juvenile library and one cold stressed library). Total RNA of each sample (about 1×10^6^ individuals) was extracted using TRI Reagent® Solution (MRC, Cincinnati, OH) according to the manufacturer's protocol. Novex 15% TBE-Urea gel (Invitrogen) was used to isolate small RNA fragments, 14–30nt in length, from total RNA. The purified small RNAs were ligated to a 5′ adaptor (Illumina, San Diego, CA, USA) and the ligation products were purified on Novex 15% TBE-Urea gel. After that, a 3′ adaptor (Illumina) was ligated to the 5′ ligation products and further purified on Novex 10% TBE-Urea gel (Invitrogen). Later, reverse transcriptase PCR (RT-PCR) was used to amplify the reverse transcribed DNAs of these ligation products. Then, 6% TBE-Urea gel (Invitrogen) was used to purify the amplification products. Lastly, DNA fragments were used for clustering and sequencing with the Illumina Genome Analyzer at the Beijing Genomics Institute, Shenzhen, China.

### Computational methods to search miRNAs and other small RNAs

**Figure 9:**
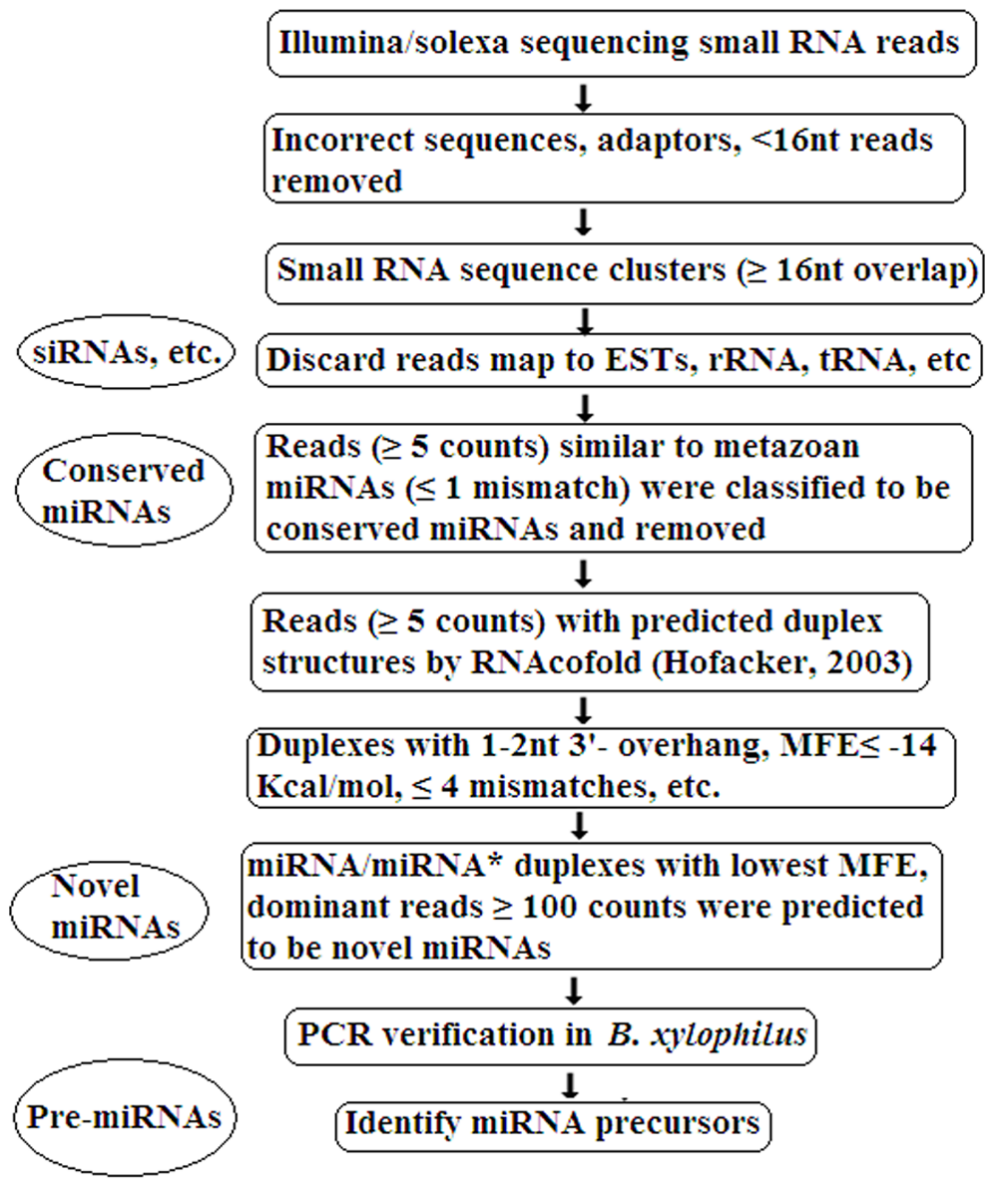
Workflow of the microRNA discovery.

The computational workflow is shown in Figure 9. First, reads from incorrect sequencing, adaptor sequences and sequences shorter than 16nt were removed. Unique sequences were retained with associated count numbers of the individual sequence reads. Second, all the clean reads were grouped into clusters based on sequence identity and a minimum of 16nt overlap length. We obtained 13,340 ESTs and 71 rRNAs of *B. xylophilus*, and 37 rRNAs of *B. mucronatus* from GeneBank (www.ncbi.nlm.nih.gov) and 1,053 known RNAs (including rRNAs, tRNAs and snRNAs) of *C. elegans* from WormBase (www.wormbase.org). Using MegaBLAST search (window size at 7nt), the sequenced reads that perfectly matched these data were removed. Last, the remaining clean sequenced reads were used to search both conserved and novel miRNAs.

Based on the consensuse that the ‘seed’ region (2–7nt in 5′ end of mature miRNA) is conserved in the same miRNA family, we used BLAST to search conserved miRNAs against the published miRNA datasets (miRBase release 14.0, September 2009) [50]. Sequenced tags of more than 5 reads that matched perfectly or near-perfectly (no more than 1 mismatch and mismatch not positioned in seed region) to metazoan mature miRNAs were assumed to be conserved miRNA candidates.

We followed the method described by Wei *et al.* [30] to predict novel miRNA/miRNA* duplexes. A pipeline was designed in Perl language to find miRNA/miRNA* duplex candidates in our small RNA libraries, combining the use of RNAcofold in the RNAfold softerware package [51]. Parameters were set as Wei *et a*l [30]), with the minimum free energy (MFE) of miRNA/miRNA* duplexes≤−14 kcal/mol; and more than 100 counts. We used the selected dominant sequences as the query dataset of potential mature miRNAs to run the Perl script pipeline against all the remaining clean sequence reads. After searching, only miRNA/miRNA* duplex candidates with the lowest MFE were retained and the paired tags belonging to the same cluster were eliminated. Sequences left were considered to be novel miRNA and miRNA* candidates.

We applied miRDeep [31] to discover miRNA precursors by combining high-throughput sequencing small RNA data with a *B. xylophilus* genome dataset generated by 454/Roche sequencing in our laboratory (unpublished), using default parameters. The small RNAs that matched perfectly with precursors and exhibited a 1–2nt 3′ overhang pattern in hairpin structures were identified as miRNA/miRNA* pairs.

We also used the RNALfold program in the RNAfold package [51] to scan the 13,340 ESTs of *B. xylophilus.* A Perl script was written to select stem-loop structures that used the following parameters: (1), MFE ≤−21 kcal/mol; (2), No multi loop; (3), both the 3′ and 5′ arms must be perfect matched with sequenced small RNAs in the stems and no overlap with the loops, and exhibit a 1–2nt 3′ overhang pattern in hairpin structures as found in many studies [15], [52]. Unfortunately, we found no candidates in the ESTs.

We used BLAST 2.2.6 [53] to search small RNAs that perfectly match the antisense strand of *B. xylophilus* ESTs. The matched small RNAs were identified to be endo-siRNAs.

Finally, Mfold [54] was used to predict the hairpin structures of precursors.

### PCR verification of miRNA precursors

We used experimental methods to verify the miRNA precursors. Genomic DNA was extracted from all stages of the nematode with a Gentra Puregene Tissue Kit (Qiagen, Valencia, CA, USA) following the manufacture's protocol. For conserved miRNA/miRNA* candidates, one primer pair was designed at the stem of each mature miRNA by aligning the miRNA sequence with metazoan miRNA precursors using Clustal W [55]. Because the novel miRNA candidates, when mature miRNAs, might locate in either arm of the precursors, we designed two primer pairs for each miRNA/miRNA* duplex. Primer3 software [56] was chosen to evaluate primers using relaxed parameters. Primers for amplification of 53 miRNA precursors are presented in supplemental Table S7.

PCR was carried out by the following scheme: 94°C, 2 min and 35 cycles (94°C, 30 s; an appropriate annealing temperature, between 50°C–55°C, 30 s; 72°C, 30 s) and a final 72°C step for 10 min. The PCR products were examined by 3.5% agarose gel electrophoresis. Fragments between 50–100nt in length were subcloned into pMD18-T vector (Takara, Dalian, Liaoning, China) for sequencing analysis.

### MicroRNA target prediction

MiRanda 3.1 [57] was used to predict microRNA targets based on 13,340 *B. xylophilus* ESTs plus a dataset of 3′UTRs derived from 74 records of *B. xylophilus* genes published in GeneBank (www.ncbi.nlm.nih.gov). The miRanda score thresholds were set to be 150 for ESTs and 50 for UTRs. ESTs predicted to contain miRNA target sites were classified using Gene Ontology (www.geneontology.org) according to the descriptions of their best hit *C. elegans* homologous genes in WormBase (www.wormbase.org) by BLAST search (e-value: 1e^−5^; ≥90% identity; ≥90nt align length).

### Real-time qPCR

A real-time qPCR was performed to compare the mRNA expression levels of two heat shock protein genes (*hsp12*, *hsp-1*) of the nematode at cold stressed status and at normal status. Total RNA was extracted from the nematodes in Trizol reagent (Invitrogen, Carlsbad, CA, USA) following the Invitrogen protocol. The total RNA was reverse transcribed to cDNA using a SuperScript™ First-Strand Synthesis System of RT-PCR kit (Invitrogen), according to the manufacturer's instructions. With cDNA as a template, quantitative PCR was carried out using the kit, TransStart SYBR Green qPCR Supermix (TransGen Biotech, Beijing, China) in a 7500 real-time system (Applied Biosystems, Carlsbad, CA, USA). The sequences of primers were listed in supplemental Table S8. A 20-*ul* reaction mixture including 1 *ul* of cDNA, 0.4 *ul* of each primer (10 µM), 10* µl* Power SYBR Green PCR Master Mix (Applied Biosystems, Warrington, UK) and 8.2* µl* H_2_O was placed in 0.2 *ml* eight-strip PCR tubes (Axygen). Cycling conditions were: 95°C for 10 min, followed by 40 cycles of 95°C for 15 s and 60°C for 10s, 72°C for 60 s. The *actin* gene of *B. xylophilus* was used for normalization of cDNA templates. Raw quantification cycle (Cq) values were calculated with the SDS software v.2.1 using manual baseline settings from 3 to 15 and a threshold of 0.2. The comparative threshold cycle (Ct) method was used for the calculation of fold changes in gene expression [58]. Three technical replications were taken for each sample. The experiment was repeated three times from RNA preparation to RT-PCR. Independent-Samples T Test was performed with the program SPSS 12.0 software [59] to test the differences between the expression levels of heat shock protein genes in the cold-shocked and the normal nematodes. The difference was statistically significant at p<0.05.

## Supporting Information

Figure S1 The predicted hairpin structures of 53 miRNA precursors. Mature miRNAs are colored in green. All the hairpin structures were predicted with Mfold.
(0.45 MB DOC)

Figure S2 Alignments of small RNA sequences matched to the 57 verified miRNA precursors in each small RNA library.
(0.48 MB XLS)

Table S1 Details of predicted 49 conserved *B. xylophilus* miRNA candidates.
(0.03 MB XLS)

Table S2 Details of predicted 761 novel *B. xylophilus* miRNA candidates.
(0.26 MB XLS)

Table S3 Details of 57 experimentally verified miRNAs and their precursors of *B. xylophilus*.
(0.03 MB XLS)

Table S4 List of predicted miRNA targets in *B. xylophilus* ESTs.
(0.50 MB XLS)

Table S5 List of predicted miRNA targets in the 3′UTRs of known *B. xylophilus* genes.
(0.04 MB XLS)

Table S6 *B. xylophilus* miRNA families, with the corresponding known miRNAs of the same seed motifs in other animals.
(0.03 MB XLS)

Table S7 List of PCR primers for miRNA precursors.
(0.03 MB XLS)

Table S8 List of Primers for RT-qPCR.
(0.01 MB XLS)
