# Supplementary material for: MicroRNA Discovery and Analysis of Pinewood Nematode Bursaphelenchus xylophilus by Deep Sequencing
Source: PLoS One. 2010 Oct 12;5(10):e13271. doi: 10.1371/journal.pone.0013271 (PMC2953492; doi:10.1371/journal.pone.0013271)
Supplement: Figure S1 — The predicted hairpin structures of 53 miRNA precursors. Mature miRNAs are colored in green. All the hairpin structures were predicted with Mfold. (0.45 MB DOC) [file pone.0013271.s001.doc]

**Supplemental Figure S1**  The predicted hairpin structures of 53 miRNA precursors. Mature miRNAs are colored in green. All the hairpin structures were predicted with Mfold [58].


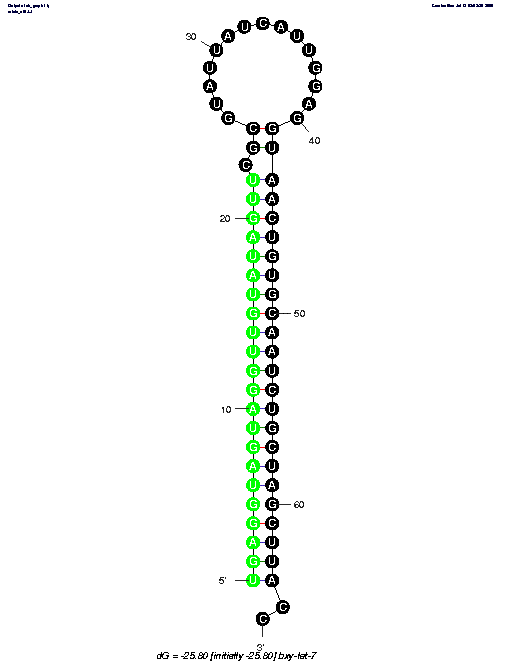

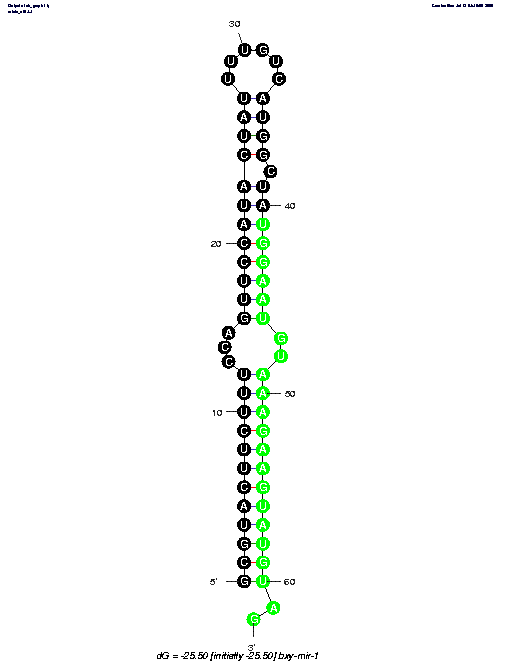

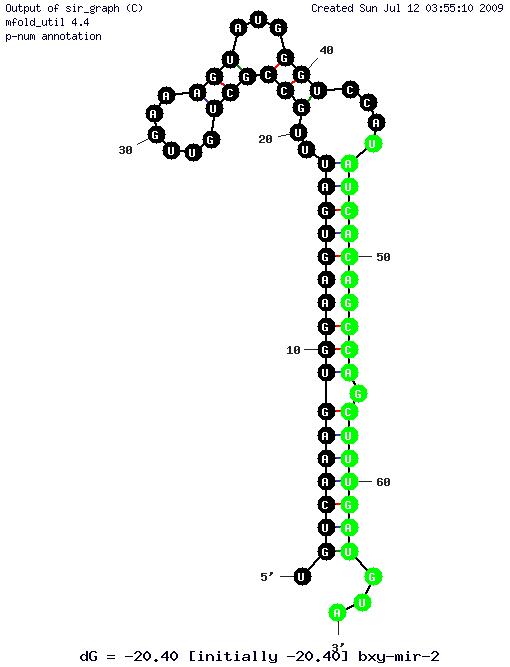

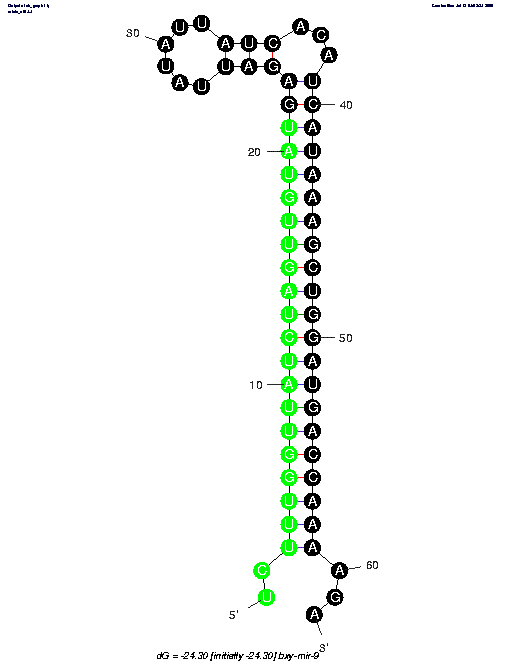

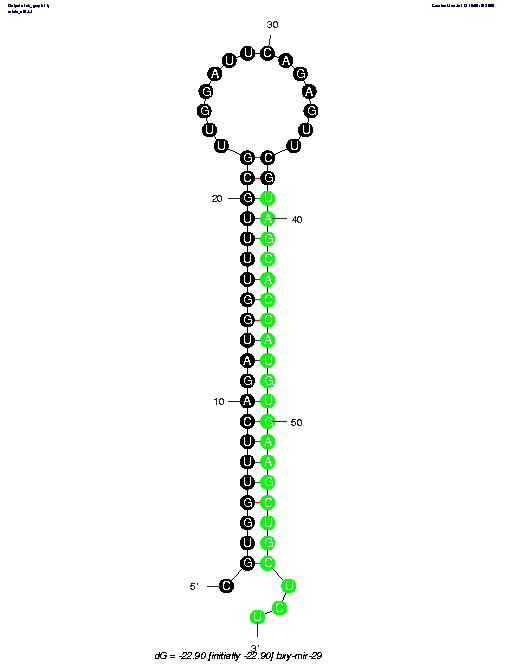

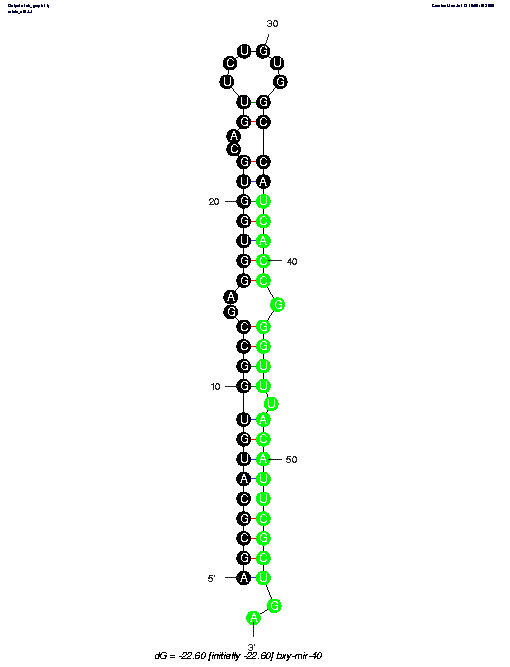

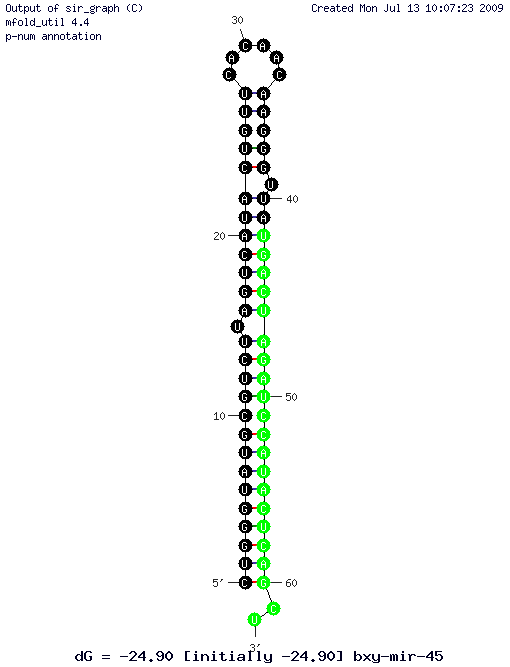

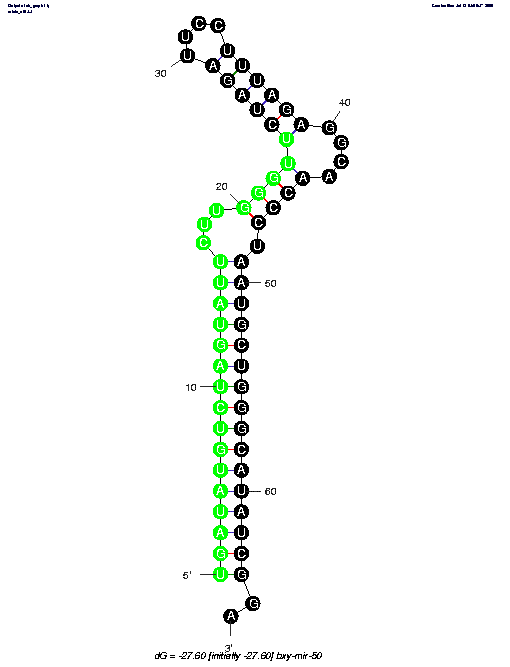

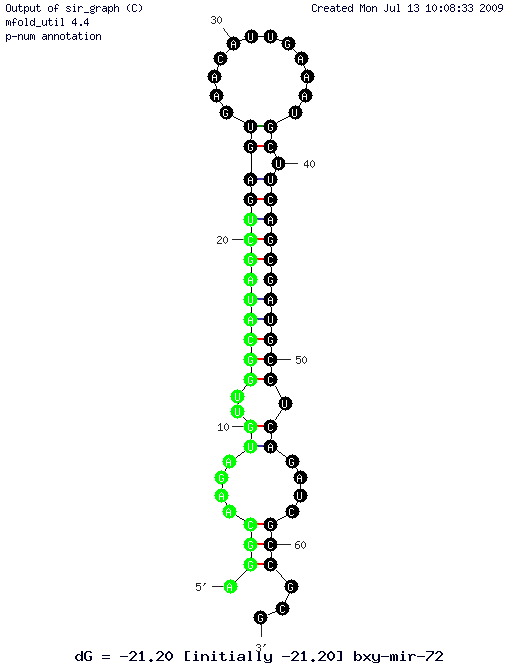

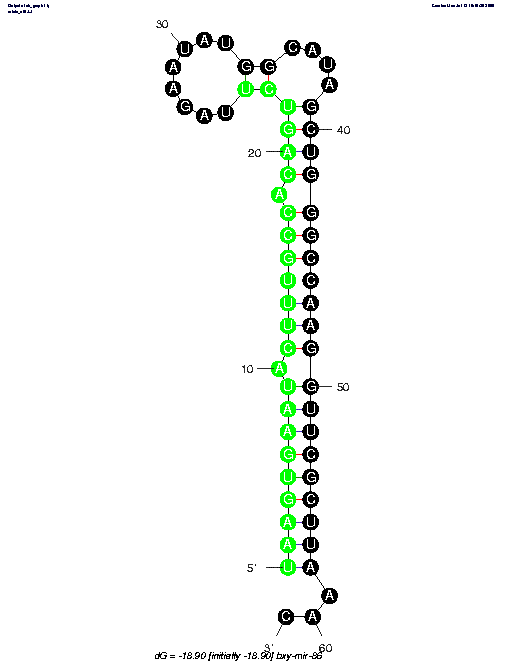

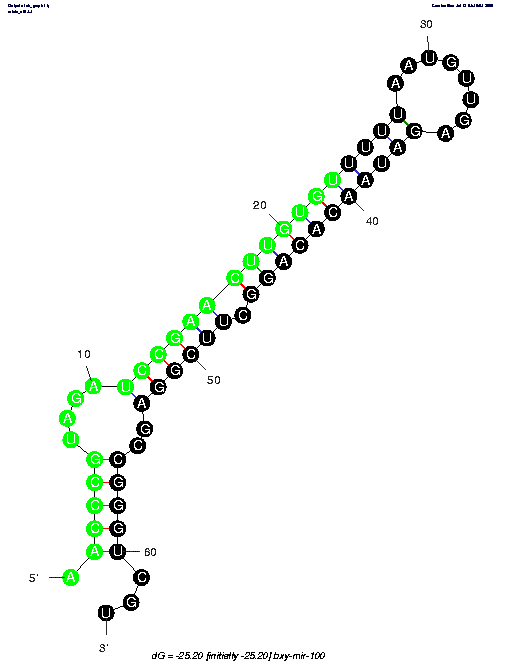

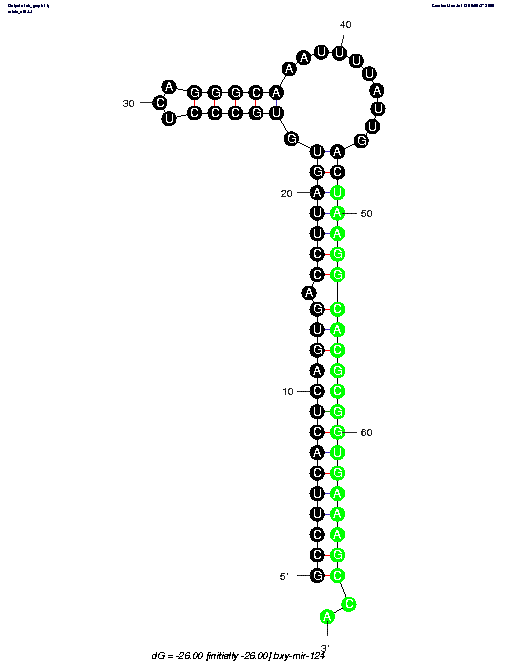

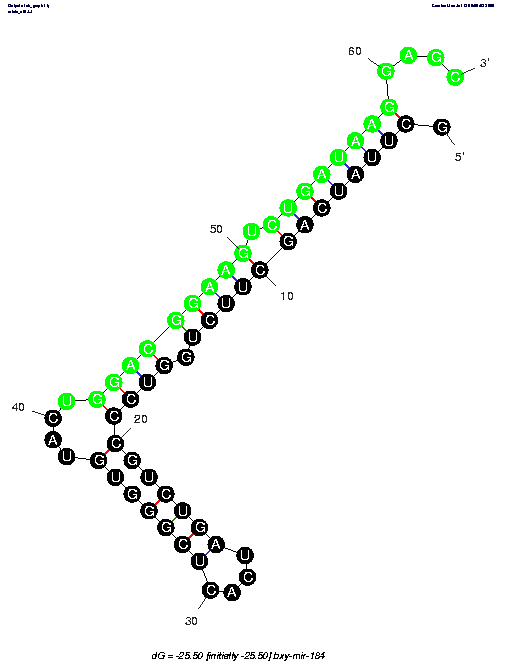

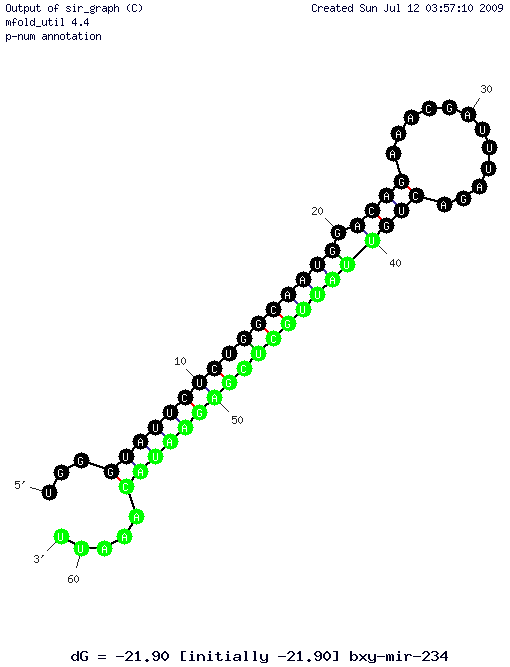

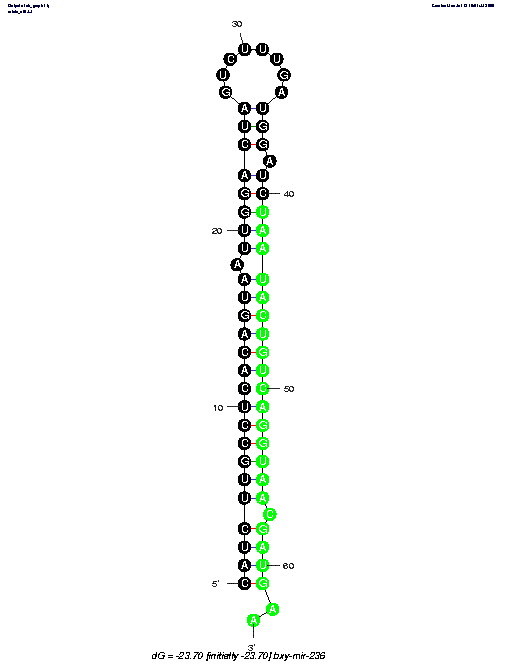

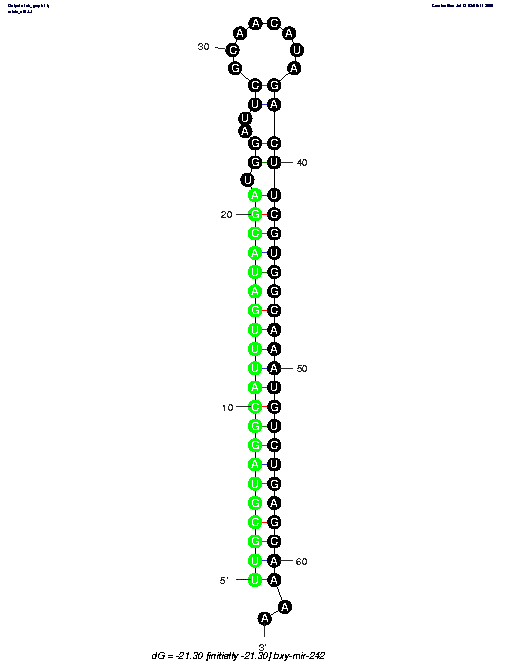

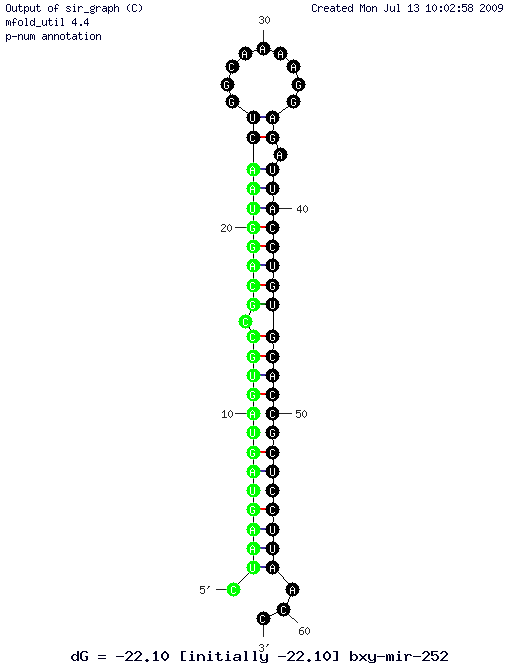

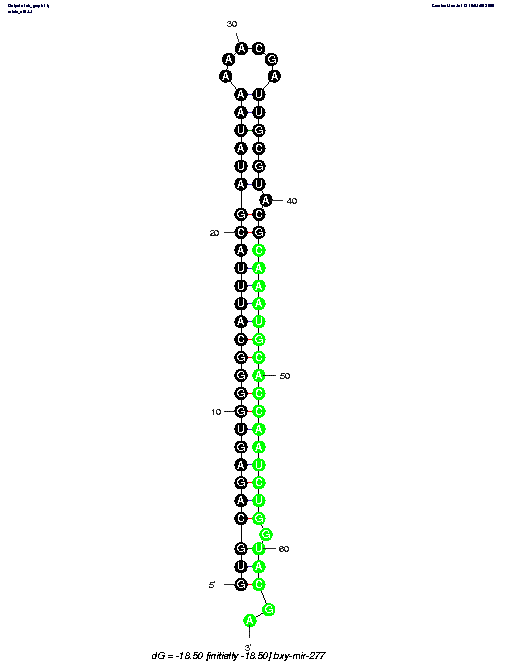

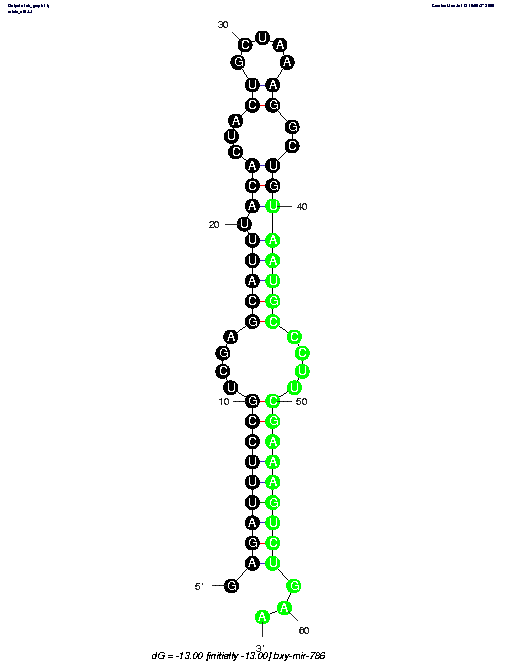

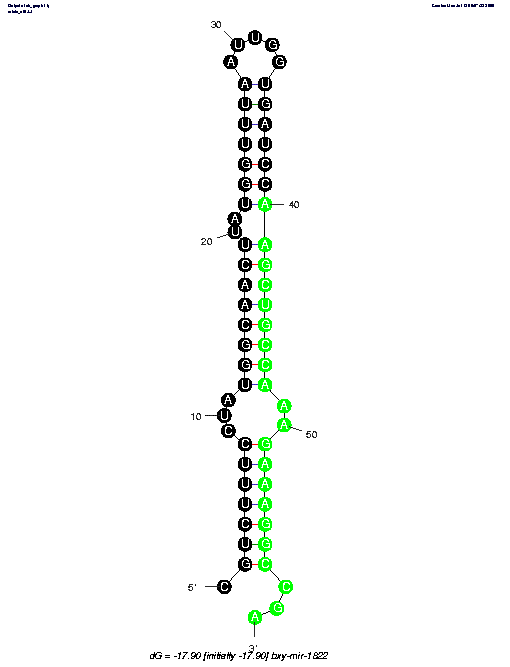

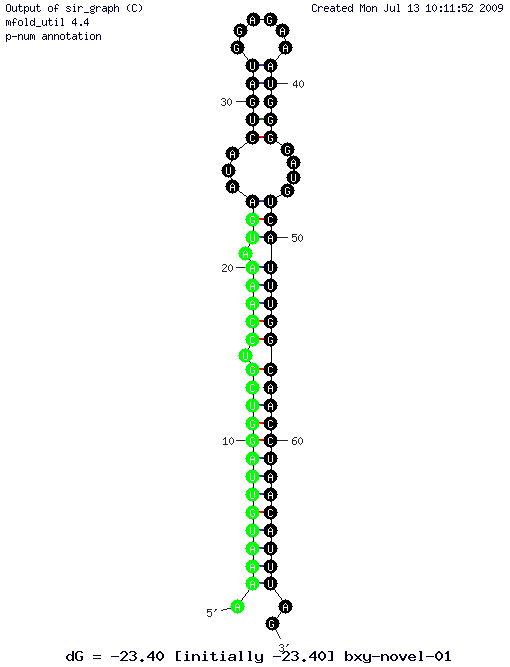

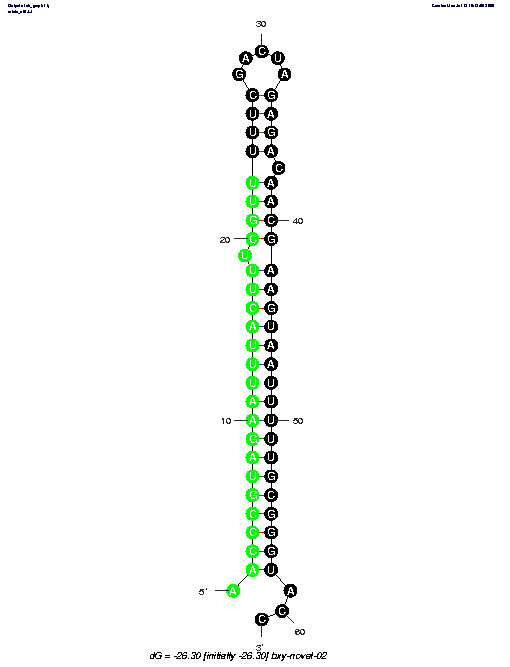

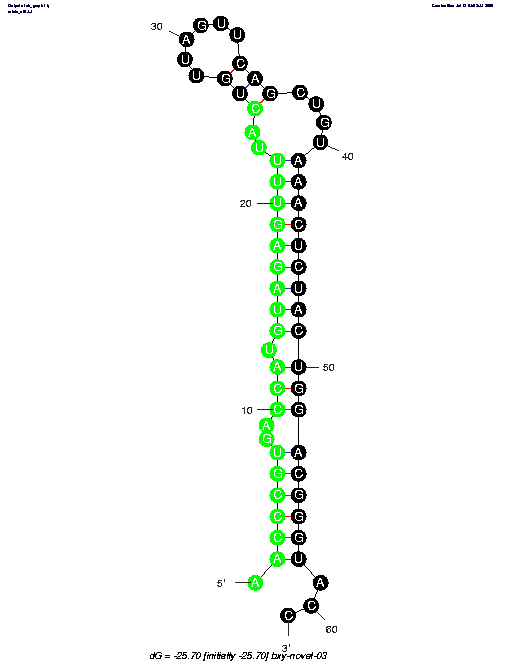

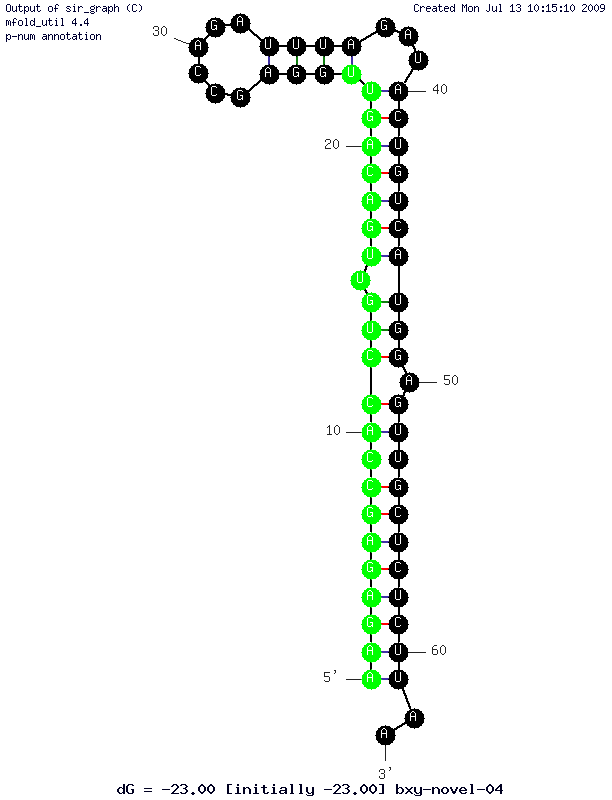

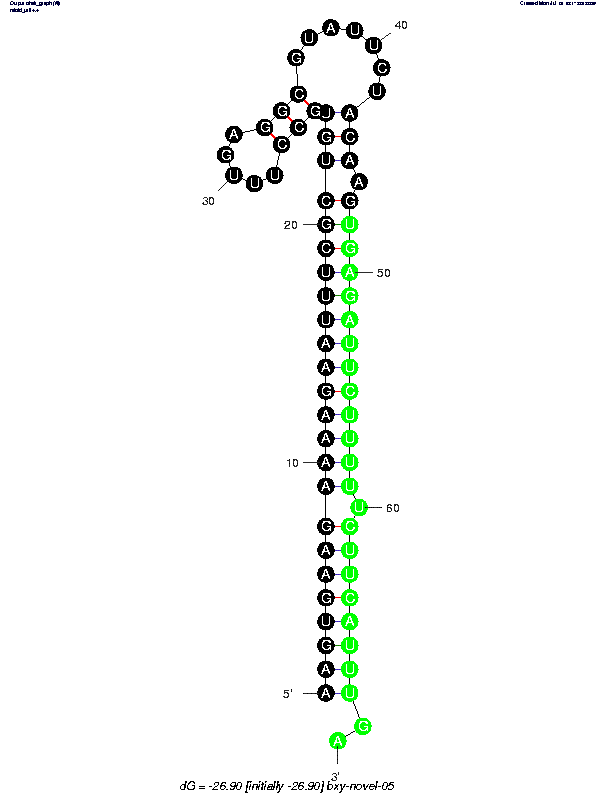

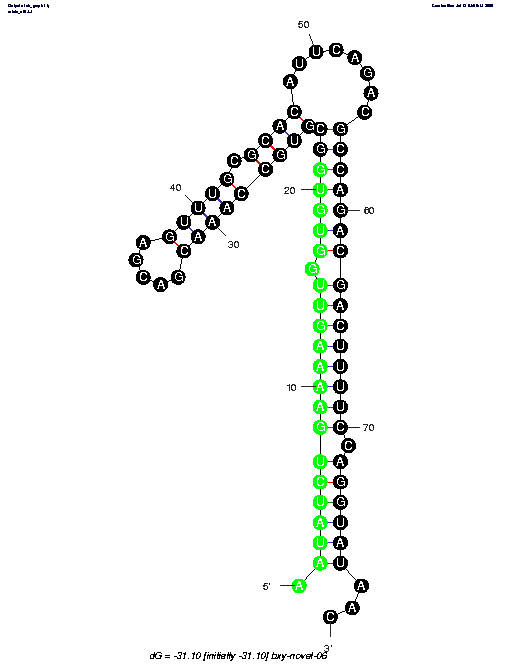

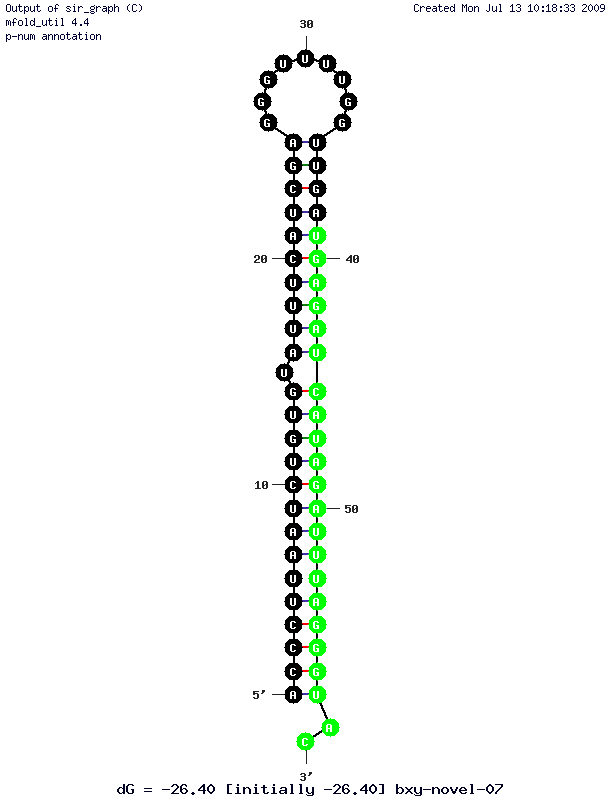

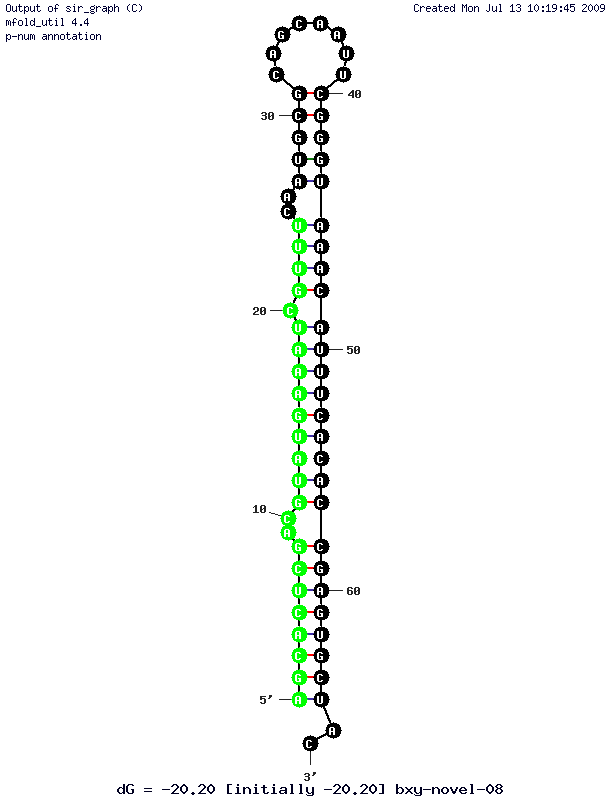

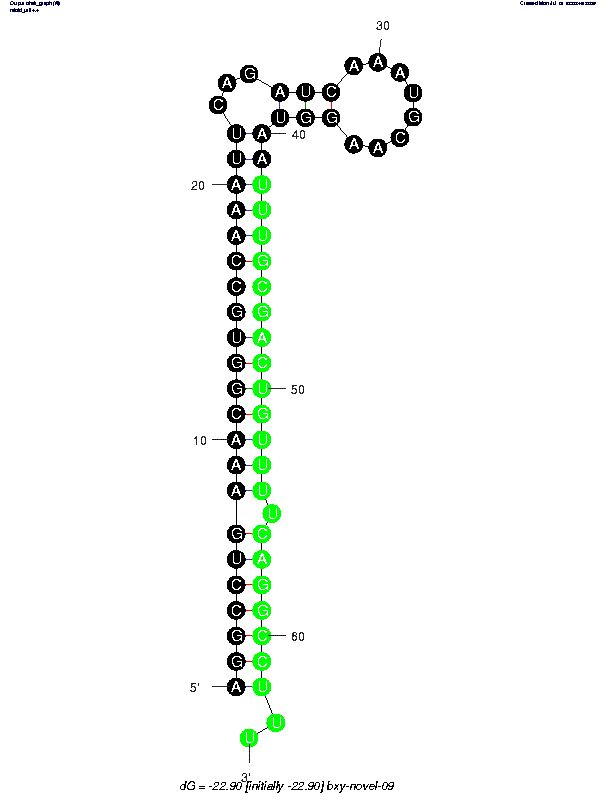

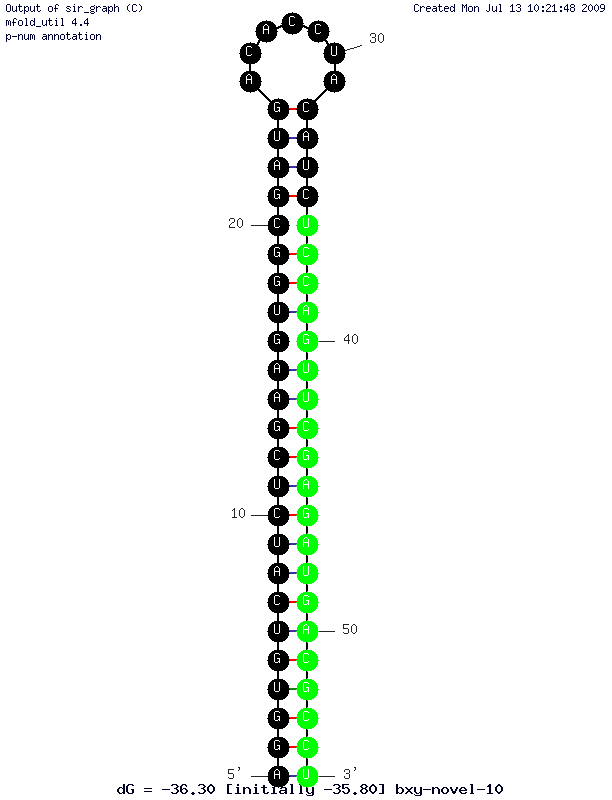

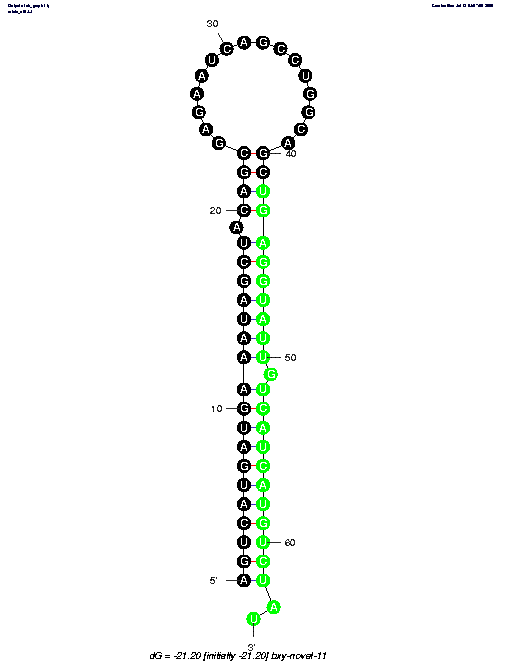

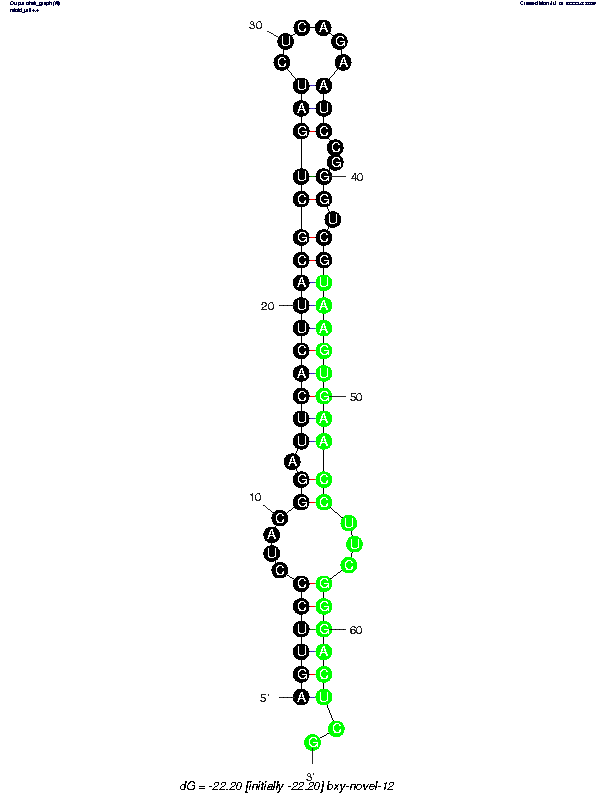

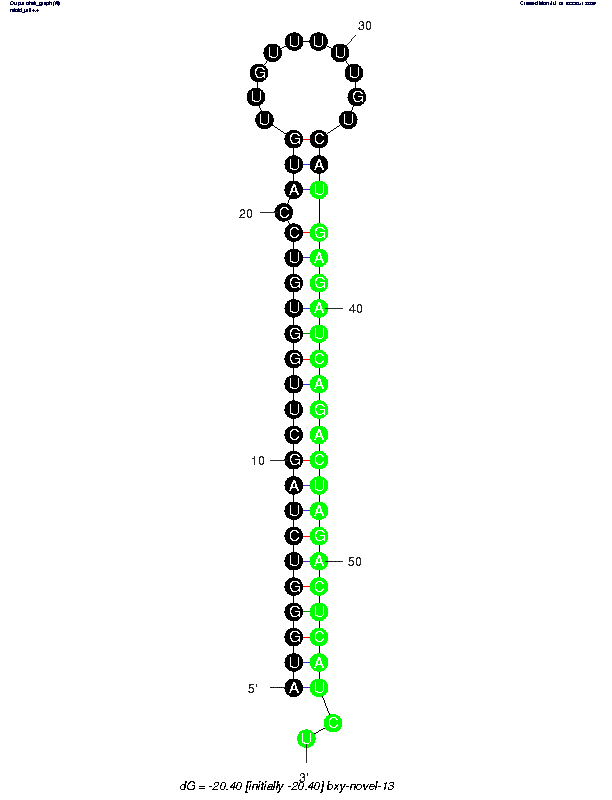

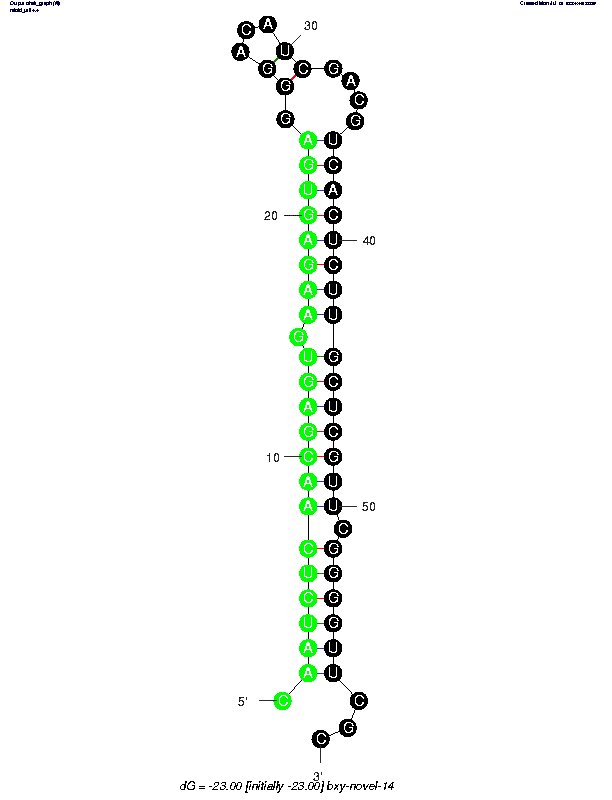

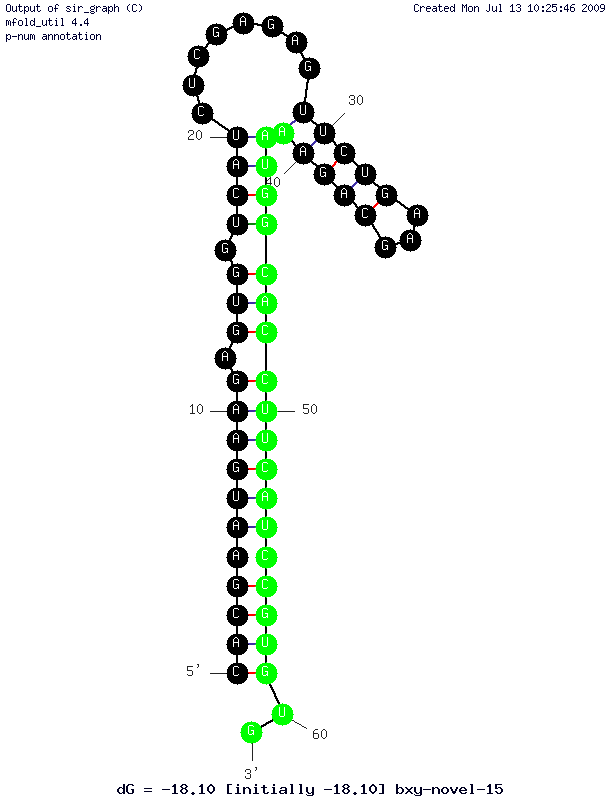

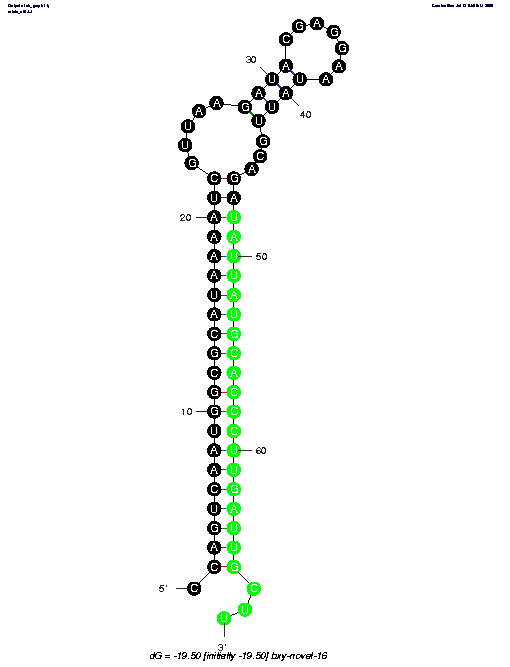

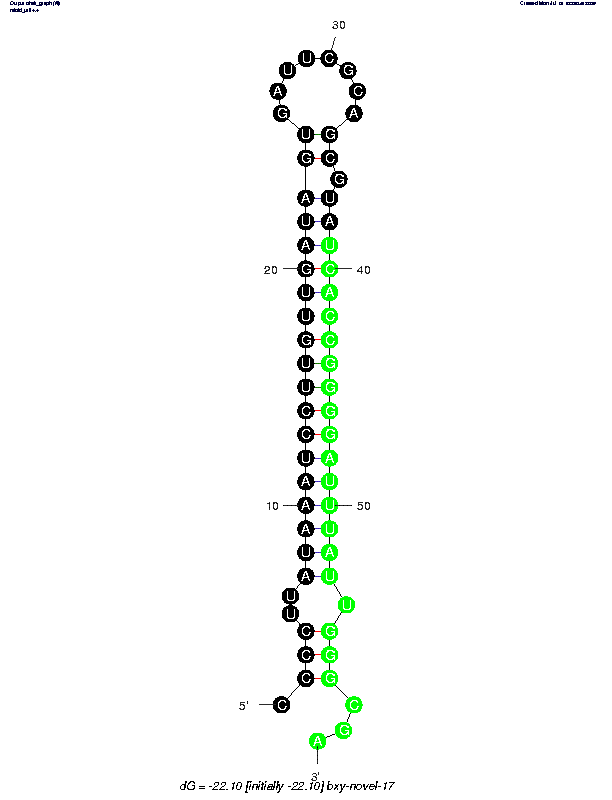

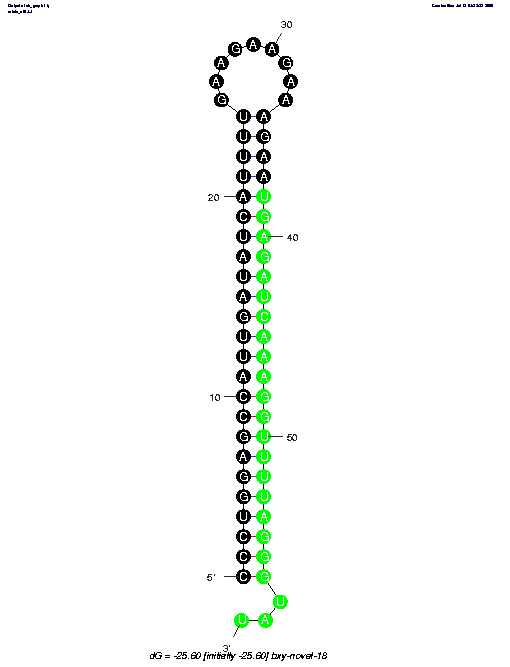

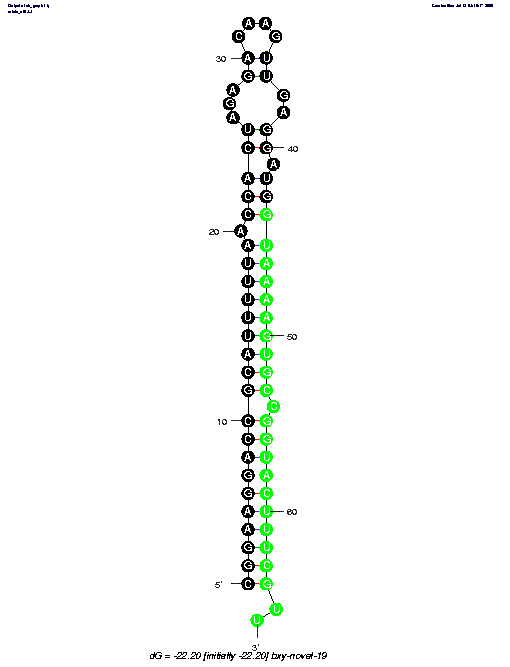

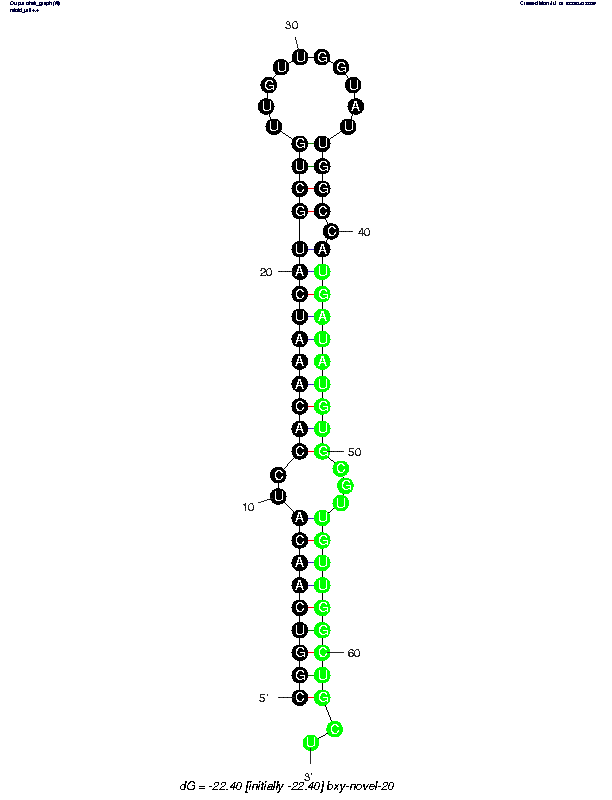

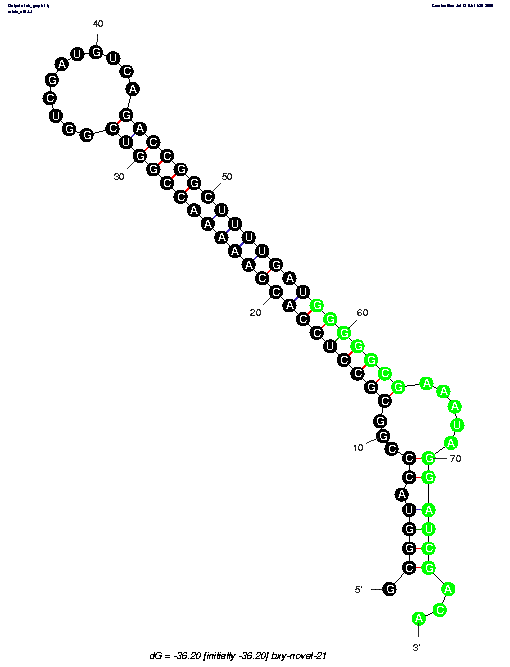

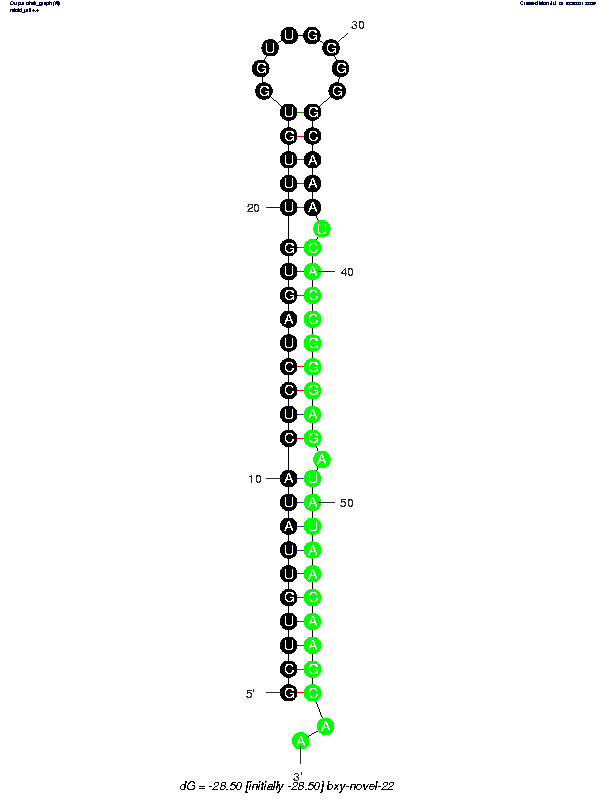

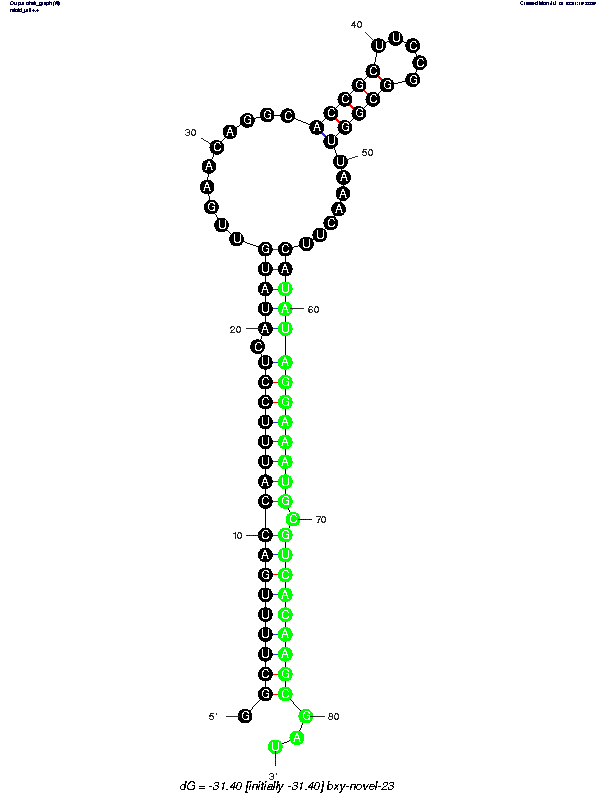

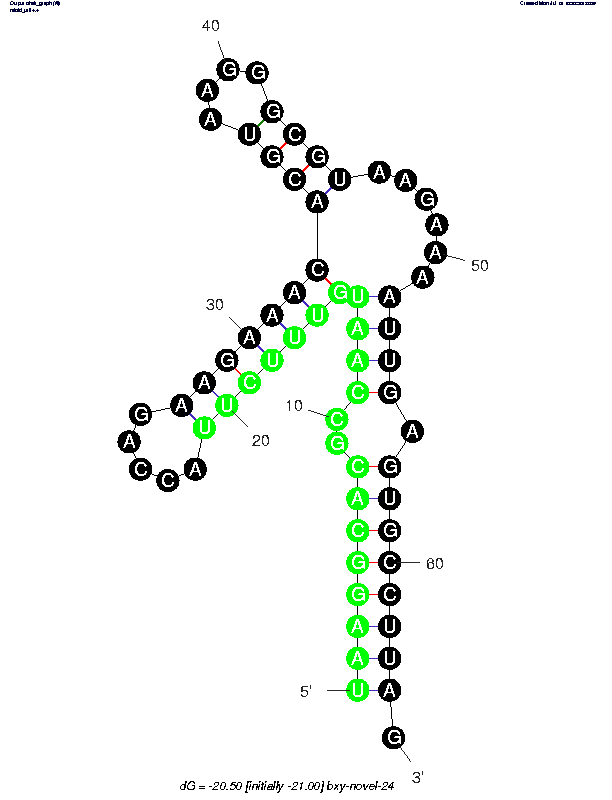

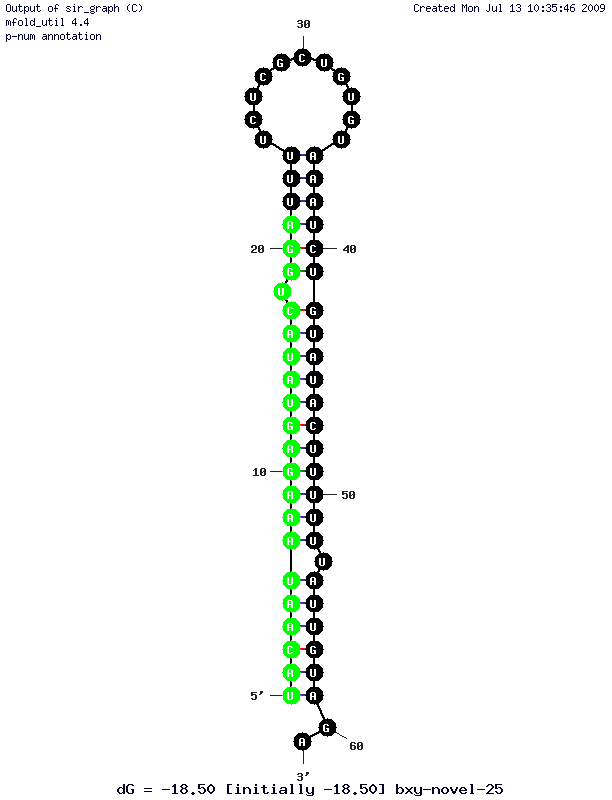

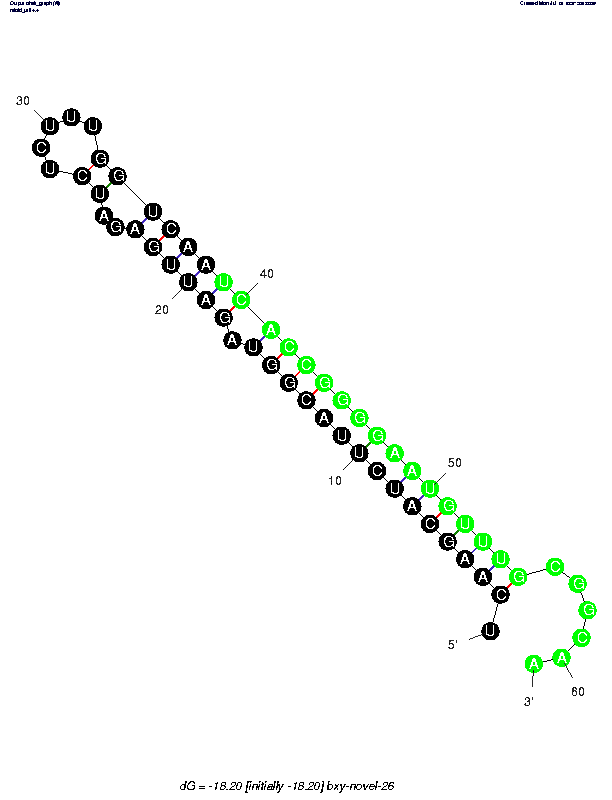

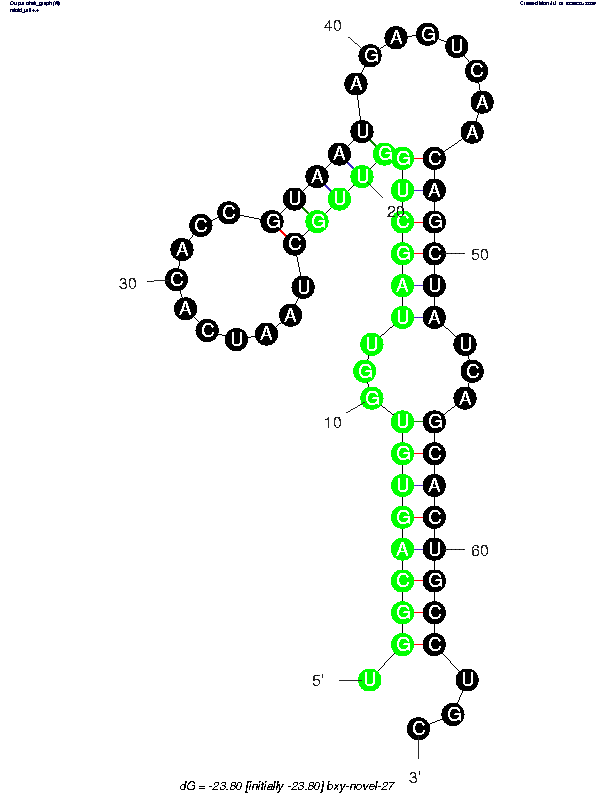

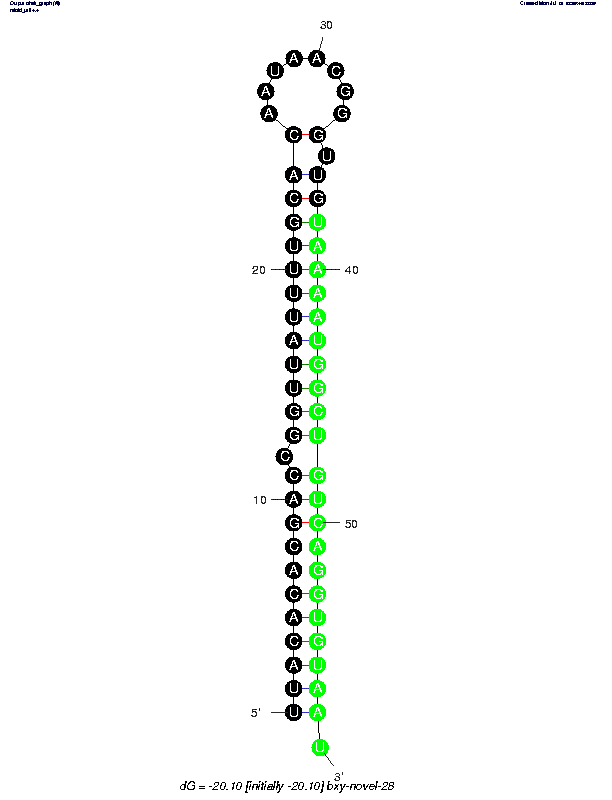

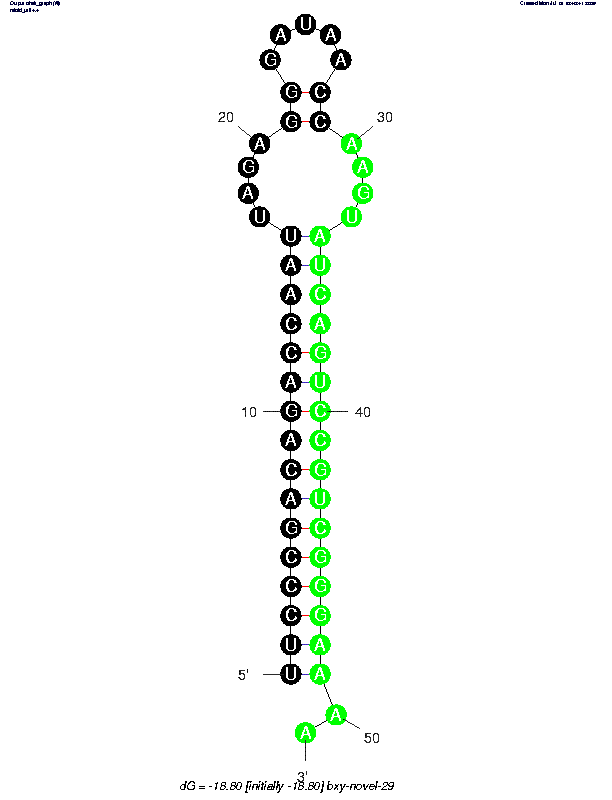

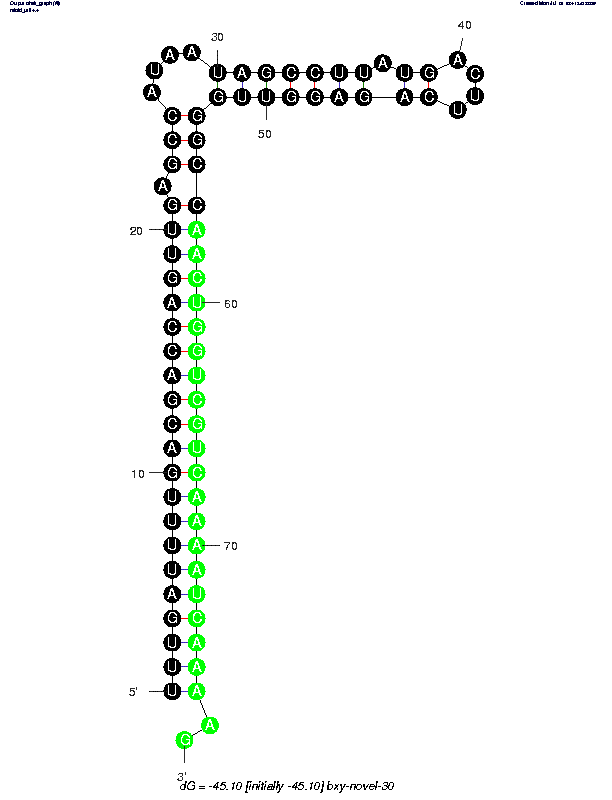

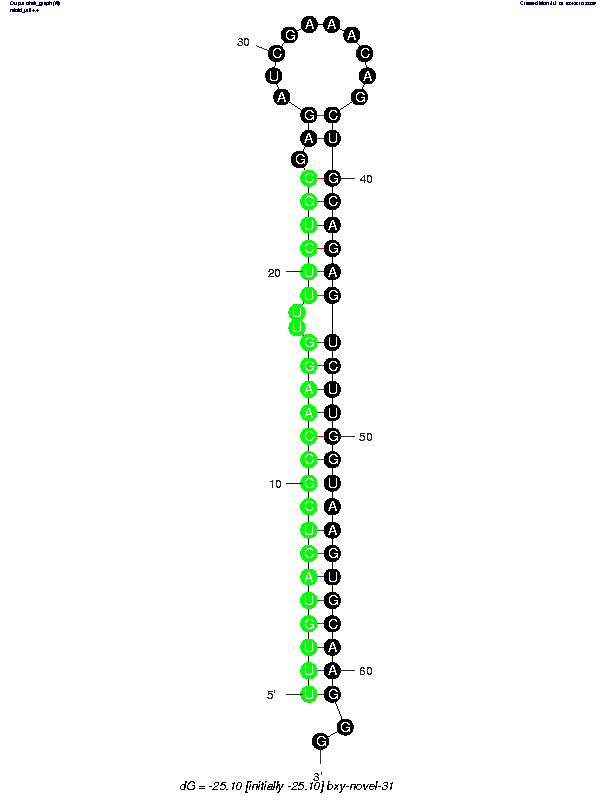

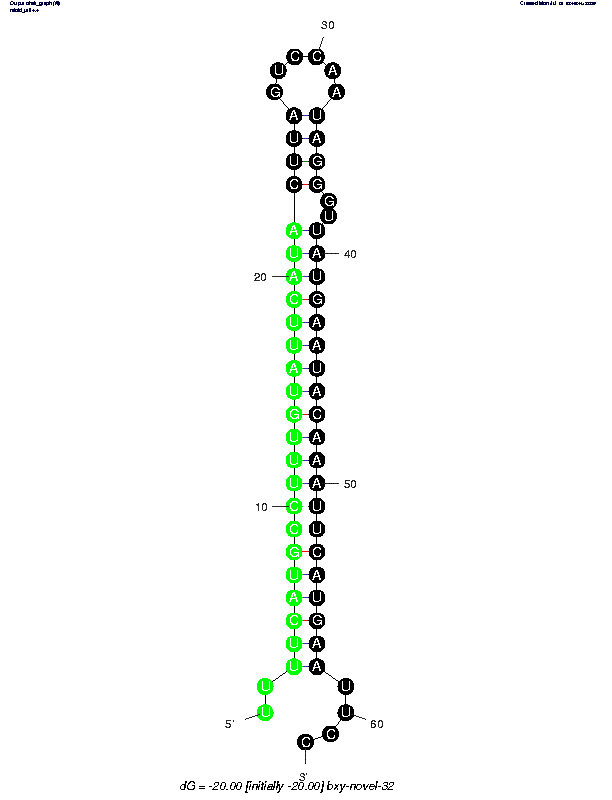

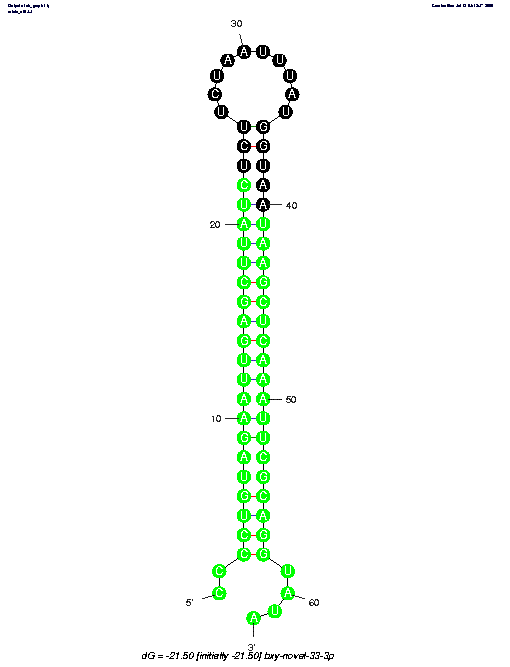


*Note*: The structure of *bxy-novel-24* depicted here is not in consistent with the previously predicted structure by RNAfold [52].
